# Kombucha Ferments from White and Red Cabbage By-Products as a Sustainable Source of Metabolites with Antioxidant and Anti-Inflammatory Activity

**DOI:** 10.3390/molecules31111886

**Published:** 2026-06-01

**Authors:** Zofia Nizioł-Łukaszewska, Aleksandra Ziemlewska, Agnieszka Mokrzyńska, Magdalena Wójciak, Ireneusz Sowa, Martyna Zagórska-Dziok

**Affiliations:** 1 Department of Technology of Cosmetic and Pharmaceutical Products, Medical College, University of Information Technology and Management in Rzeszow, Sucharskiego 2, 35-225 Rzeszow, Poland; zniziol@wsiz.edu.pl (Z.N.-Ł.); aziemlewska@wsiz.edu.pl (A.Z.); amokrzynska@wsiz.edu.pl (A.M.); 2 Department of Analytical Chemistry, Medical University of Lublin, Aleje Raclawickie 1, 20-059 Lublin, Poland; magdalena.wojciak@umlub.pl (M.W.); ireneusz.sowa@umlub.pl (I.S.)

**Keywords:** cabbage, kombucha ferments, antioxidants, cytotoxicity assessment, anti-inflammatory activity, antibacterial activity

## Abstract

Cabbage (*Brassica oleracea* var. capitata) is a widely cultivated vegetable rich in bioactive compounds, but its industrial processing generates significant underutilized by-products, especially cabbage cores. This often-discarded fraction represents a promising and sustainable source of valuable functional components with potential applications in food systems. The study evaluated the biological activity of extracts and kombucha ferments obtained from the leaves and cores of white and red cabbage. Antioxidant capacity was assessed using ABTS, DPPH, and FRAP assays, while intracellular reactive oxygen species (ROS) levels were evaluated in a cell-based model. Cytotoxicity was determined using fibroblast and keratinocyte cell lines with Alamar Blue and Neutral Red assays. Anti-inflammatory activity was assessed by measuring the levels of the cytokines (IL-1β and IL-6) using ELISA, and antimicrobial effects were tested against bacteria associated with skin inflammation. The results showed that fermented white and red cabbage extracts have stronger antioxidant, anti-inflammatory, and antimicrobial effects than unfermented extracts, with the best results observed after 20 days of fermentation. Low concentrations have a beneficial effect on skin cell viability, while higher concentrations result in reduced viability. These results highlight the potential of kombucha-fermented cabbage leaves and cores as a versatile and sustainable source of bioactive compounds for health-promoting applications.

## 1. Introduction

Cabbage (*Brassica oleracea* var. capitata), particularly the white and red cabbage varieties, is a widely cultivated vegetable and a significant source of active ingredients. Due to its high content of bioactive compounds and good availability of raw materials, cabbage is an important raw material for further processing. However, during industrial processing and preparation of the raw material for distribution, a large amount of by-product biomass is generated, a significant fraction of which is the cabbage core. Unlike the leaves, which are commonly used as a food product, the core is most often discarded as process waste. Furthermore, given the need for the efficient management of plant materials generated during cabbage processing, this fraction may constitute an underutilized source of bioactive compounds and a potential source for obtaining components of high functional value [1,2,3,4].

Cabbage, both white and red, is a source of a wide spectrum of compounds with antioxidant, anti-inflammatory, and antibacterial properties. The most important of these are glucosinolates and their degradation products, such as isothiocyanates and indoles, which modulate oxidative stress pathways and inflammatory responses at the cellular level. Cabbage is also a significant source of phenolic compounds, primarily hydroxycinnamic acids, such as ferulic, caffeic, and p-coumaric acids, as well as flavonols, such as quercetin and kaempferol derivatives [5,6,7,8]. In the case of red cabbage, a key antioxidant fraction consists of anthocyanins, such as cyanidin-3-diglucoside-5-glucoside [9,10]. Another crucial aspect of cabbage’s bioactivity is its antibacterial properties, primarily related to the presence of isothiocyanates formed by the hydrolysis of glucosinolates, such as sulforaphane and phenethyl isothiocyanate, which have the ability to inhibit the growth of Gram-positive and Gram-negative bacteria [11,12,13]. The biological properties of white and red cabbage mean that they can be treated as sources of functional plant components of application importance, intended for further processing.

The fermentation process of cabbage, in which lactic acid bacteria play a dominant role, is a crucial step in the biotransformation of its components, leading to partial degradation of the cell wall, release of phenolic compounds, and transformation of glucosinolates [14,15]. These changes may affect the bioavailability and biological activity of the resulting metabolites, including those with antioxidant, anti-inflammatory, and antimicrobial properties. Broadly speaking, fermentation of plant materials is currently viewed as a green process that combines the functions of extraction and biotransformation, and a growing number of studies indicate its role in the structural modification and bioavailability of phytochemicals [16,17,18].

In this context, fermentation with a symbiotic SCOBY (Symbiotic Culture of Bacteria and Yeast) of yeast and acetic acid bacteria (kombucha) represents an extension of classical fermentation processes. It involves a complex consortium of yeasts and acetic acid bacteria, which leads to the biotransformation of phenols and the production of a broad profile of organic acids, simultaneously enhancing the acidifying effect and antimicrobial properties [19,20,21,22]. Besides traditional raw materials such as black and green tea, a growing number of studies indicate that fermentation with a symbiotic SCOBY of yeast and acetic acid bacteria can occur with various plant raw materials, including fruits and fruit juices, leading to the creation of products with diverse phenolic profiles and antioxidant properties. In recent years, studies have also begun to appear on the fermentation of vegetables using kombucha cultures. It has been shown, for example, that the fermentation of carrots, celery, and parsley significantly affects the content of phenolic compounds, free radical scavenging activity, and anti-inflammatory properties. These reports highlight kombucha fermentation as a versatile process combining the extraction and biotransformation of bioactive plant components. The fermentation of white and red cabbage, including waste fractions, with kombucha may represent a promising green technology leading to the creation of new mixtures of bioactive metabolites with potential industrial significance [23,24,25].

Although cabbage fermentation has been extensively described in the literature, previous studies have mainly focused on traditional lactic acid fermentation processes and commonly consumed cabbage tissues intended for food applications [26,27]. In contrast, data regarding the use of SCOBY-based kombucha fermentation for cabbage-derived materials remain very limited. Moreover, outer cabbage leaves and cores are generally treated as low-value by-products generated during food processing and are frequently discarded despite their considerable content of biologically active compounds. In the present study, outer cabbage leaves and cores were applied as substrates for kombucha fermentation and evaluated as potential sources of multifunctional bioactive metabolites. Therefore, this work combines two important aspects: the application of an unconventional SCOBY-mediated fermentation system and the valorization of underutilized cabbage by-products with potential cosmetic and biological relevance.

The aim of the conducted analyses was to compare the content of biologically active compounds and biological properties in the leaves and cores of white and red cabbage, as well as their ferments obtained after 10 and 20 days of fermentation with kombucha tea. During the study, active compounds were determined using liquid chromatography coupled to mass spectrometry. The antioxidant properties of the samples were assessed using DPPH, ABTS, and FRAP assays, and intracellular free radical (ROS) levels were measured in keratinocyte cell lines (HaCaT) and human dermal fibroblast (HDF). Cytotoxicity against these cells was determined using Alamar Blue and Neutral Red assays. Furthermore, the expression of pro-inflammatory cytokines, such as interleukin-6 (IL-6) and interleukin-1β (IL-1β), was determined, and the antibacterial properties of the extracts and ferments were assessed.

## 2. Results and Discussion

### 2.1. UHPLC–DAD–MS Analysis

The phytochemical profile of ionizable phenolic metabolites in the water extract from different cabbage cultivars was poor and was similar across all analyzed parts, including the core and leaves. Representative chromatograms are shown in the Supplementary Material (Figure S1). All the extracts contained a kaempferol derivative with the molecular formula C_27_H_29_O_16_. In some extracts of red cabbage, p-coumaroylquinic acid was detected. A clear modification of the phytochemical profile was observed in the fermented samples compared to the corresponding extracts. Fermentation resulted in the appearance and/or marked enrichment of numerous compounds belonging to phenolic acids such as galloylquinic acids, chlorogenic acids, and coumaroylquinic acids as well as flavonoids, including apigenin, quercetin, and kaempferol derivatives and catechin. The detailed chromatographic and spectrometric data used for compound identification are presented in Table S1 (Figure S2).

Quantitative analysis demonstrated that the fermented samples were significantly richer in the analyzed phenolic compounds than the non-fermented extracts, and their concentrations increased with fermentation time. The highest levels of phenolic acids and flavonoids were consistently observed in the 20-day fermented samples (Table 1 and Table 2). All cabbage varieties exhibited a broadly similar qualitative profile of phenolic acids and flavonoids; however, clear quantitative differences were observed depending on cabbage type and plant part. For example, the core of white cabbage showed higher concentrations of gallic acid compared to the core of red cabbage. Catechin and epicatechin levels were significantly higher in the fermented white cabbage core. Overall, these results confirm that both cabbage variety and fermentation duration significantly influence the quantitative composition of phenolic acids and flavonoids. This observation aligns with previous findings that fermentation can transform and enhance plant phenolic profiles, often increasing total phenolic content and antioxidant activity through microbial biotransformation of hydroxycinnamic and flavonoid compounds [28].

Because fermentation can influence the amino acid profile, we also examined the levels of free amino acids in the extracts and their bioferments. Free amino acids may have beneficial effects on the skin, as they are components of the skin’s natural moisturizing factor (NMF), serve as building blocks or precursors for structural proteins and antioxidants, and modulate biochemical pathways important for barrier function, repair, and resilience [29,30]. However, in the aqueous extracts, the levels of free amino acids were very low and, for most compounds, did not exceed 0.01 µg/mL. The most abundant amino acids were proline, valine, isovaline, aspartic acid, glutamic acid, and glutamine. Additionally, trace amounts of sulfur-containing amino acids, i.e., cysteine and methionine, were also present. For the majority of amino acids, their concentrations increased as a result of fermentation, likely due to microbial proteolytic activity and the release of amino acids from proteins and peptides (Figure S2, Tables S2 and S3). Nevertheless, exceptions were observed, as the levels of certain amino acids decreased after fermentation. Such reductions may be attributed to their further metabolic conversion by fermenting microorganisms, including transamination, decarboxylation, or incorporation into microbial biomass and secondary metabolites. Similar fluctuations in free amino acid profiles during fermentation have been reported in the literature [31,32].

From a cosmetic perspective, simple organic acids such as citric, malic, and lactic acids play also an important role due to their multifunctional properties. Citric and malic acids, both alpha-hydroxy acids (AHAs), gently exfoliate the skin, improve texture, and help maintain the optimal pH balance of formulations, supporting the skin’s protective barrier. Lactic acid, in addition to exfoliating, also enhances skin hydration and can stimulate collagen renewal [33,34]. Similarly to amino acids, fermentation was found to have a significant impact on their content (Figure S3). Tables S3 and S4 present the quantitative profiles of selected low-molecular-weight organic acids in three cabbage varieties. Fermentation led to a clear increase in organic acid content in all varieties, particularly citric and malic acids, with the highest levels generally observed after 20 days of fermentation. In addition, a significant increase in gluconic acid was detected, resulting from glucose oxidation following sugar hydrolysis. The observed increases in lactic, malic, and citric acids are consistent with previously reported organic acid profiles of fermented cabbage products, including sauerkraut, and confirm that both cabbage variety and fermentation time influence organic acid composition [35].

It should also be considered that prolonged aqueous extraction and ultrasound treatment may influence the stability of certain phytochemicals, particularly highly sensitive phenolic compounds and anthocyanins. This may partially explain the relatively low abundance of some metabolites observed in the non-fermented extracts. Nevertheless, fermentation markedly increased the content of several detected compounds and enhanced biological activity, suggesting that SCOBY-driven fermentation promoted the release or biotransformation of bioactive metabolites.

### 2.2. Assessment of Antioxidant Activity

#### 2.2.1. Antioxidant Potential Measured by ABTS, DPPH, and FRAP Assays

To obtain a broader characterization of the antioxidant potential of the analyzed extracts and ferments, three complementary assays—DPPH, ABTS, and FRAP—were applied [36]. Since antioxidant activity may depend on different reaction mechanisms, the use of multiple analytical methods allows for a more comprehensive evaluation of the samples. The DPPH assay primarily reflects the ability of compounds to scavenge stable free radicals, whereas the ABTS method can detect both hydrophilic and lipophilic antioxidant activity [36,37,38]. In turn, the FRAP assay evaluates the reducing capacity of the samples by measuring their ability to reduce Fe^3+^ ions to Fe^2+^ [39]. The combination of these assays made it possible to assess not only radical scavenging activity but also the reducing potential of metabolites present in cabbage extracts and ferments. To assess the antioxidant activity of the investigated extracts and ferments more reliably, the kombucha starter solution alone was also analyzed in parallel in order to determine its potential contribution to the observed biological effects (Figure S4). The DPPH method relies on the ability of antioxidants to reduce the stable DPPH radical, which results in a decrease in absorbance and allows for the determination of the percentage of radical scavenging [37]. The ABTS method involves the formation of the stable cationic radical ABTS by reacting ABTS with potassium persulfate, followed by measuring the degree of discoloration in the presence of antioxidants [38]. The FRAP method, on the other hand, assesses the reducing power of samples and relies on the ability of antioxidant compounds to reduce Fe^3+^ ions to Fe^2+^ [40]. By combining the DPPH, ABTS, and FRAP methods, it was possible to compare the antioxidant activity of SCOBY extracts and ferments from different parts of cabbage, taking into account the different antioxidant reaction mechanisms [34,35,36].

The studies were conducted for four sample concentrations: 50, 100, 250, and 500 µg/mL. The obtained results confirm that the analyzed samples contain antioxidant compounds capable of neutralizing free radicals. This effect can be attributed primarily to the presence of polyphenols and glucosinolates, and in the case of red cabbage, additionally to the participation of anthocyanins, which significantly influence antioxidant activity [41,42].

In both ABTS experiments carried out for white and red cabbage it was observed that free radical scavenging increases with concentration. A comparison of the parts of the plant suggest slightly more promising results for the leaves against the core. In both cases, white and red cabbage, the values registered for leaves overall were higher than for the core. When comparing white and red cabbage then for both the core and leaves respectively one can observe higher values denoted for red cabbage [43].

Another interesting observation is that fermentation has positive effect on antioxidant properties. The values observed for F10 ferments are higher than for the extract, and for F20 ferments are even higher than for F10 ferments. This observation holds within each combination of white/red cabbage, plant part and concentration. The only exception is white cabbage core with the lowest concentration, but this observation remains valid for all other combinations.

**Figure 1:**
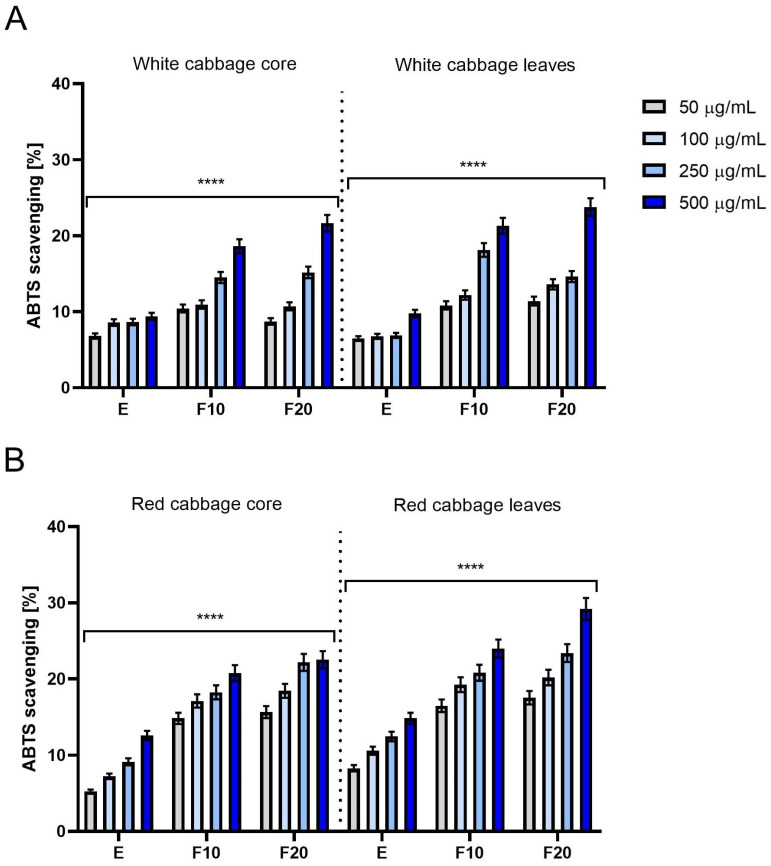
The ability of extracts (E) and ferments (F10 and F20) obtained from the core and leaves of white cabbage (**A**) and from the core and leaves of red cabbage (**B**) to scavenge ABTS free radicals at concentrations of 50, 100, 250 and 500 µg/mL. The data are presented as mean ± SD from three independent experiments, with each sample tested in triplicate **** *p* < 0.0001.

The most promising results among all the cases were obtained for red cabbage leaf, with F20 ferments in the highest concentration (Figure 1).

The results obtained from the DPPH assay demonstrate a high degree of consistency with the trends observed in the ABTS tests, further validating the antioxidant potential of the studied samples. For white and red cabbage it was observed that free radical scavenging increases with concentration. Comparisons of each part of the plant depend on the concentration, but when focusing on higher concentrations, then the results for the leaves were better than for the core. In the two lower concentrations the conclusion was the opposite; the leaves resulted in lower antioxidant properties compared to the core. The lower antioxidant activity observed for the leaf samples at lower concentrations may be related to differences in the qualitative composition of antioxidant compounds and their interactions within the extracts. Although leaves are typically richer in phenolic compounds and anthocyanins, some metabolites may exhibit limited activity at low concentrations or may respond differently depending on the assay mechanism [8,44]. In contrast, compounds present in the core may remain more active under diluted conditions. At higher concentrations, the antioxidant potential of the leaf samples became more pronounced, likely due to the higher accumulation of polyphenols and anthocyanins in leaf tissues and possible synergistic interactions between metabolites, which may enhance antioxidant activity [8,44,45].

When comparing white and red cabbage then for both the core and leaves respectively one can observe higher values denoted for red cabbage.

The effect of fermentation was positive; all the measurements increased with fermentation time for each combination of white/red cabbage, plant part and concentration. The only exception is white cabbage leaf in the concentration of 50 µg/mL and red cabbage core in the concentration of 100 µg/mL; the above statement remains valid for all other combinations.

**Figure 2:**
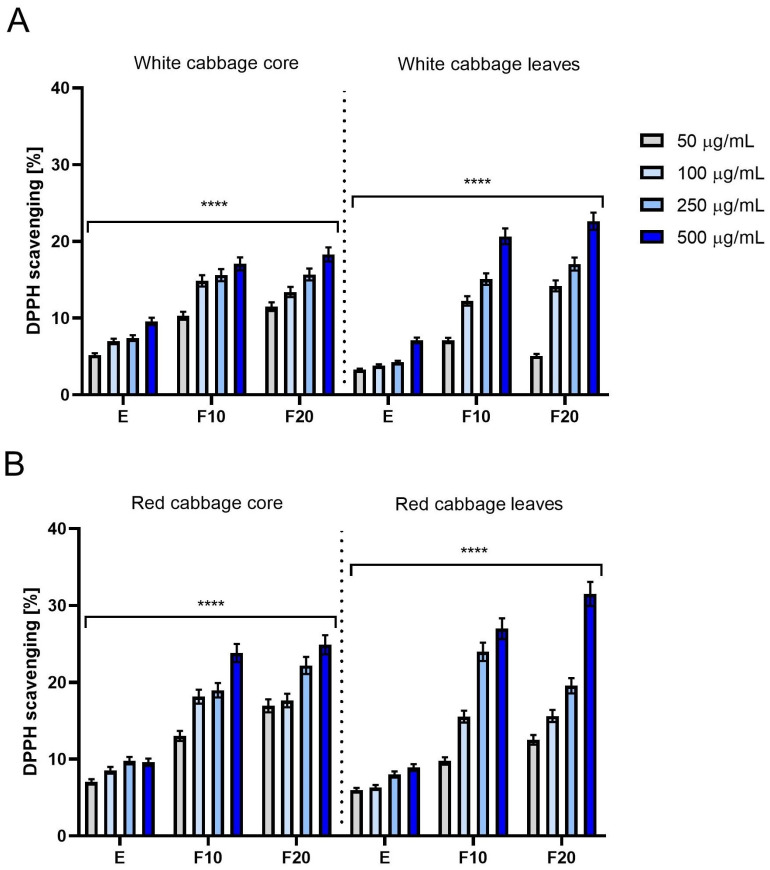
The ability of extracts (E) and ferments (F10 and F20) obtained from the core and leaves of white cabbage (**A**) and from the core and leaves of red cabbage (**B**) to scavenge DPPH free radicals at concentrations of 50, 100, 250 and 500 µg/mL. The data are presented as mean ± SD from three independent experiments, with each sample tested in triplicate **** *p* < 0.0001.

The most promising results among all cases were obtained for red cabbage leaf, F20 ferments in the highest concentration (Figure 2).

In the FRAP experiments, for white and red cabbage the same behavior as previously was observed, that free radical scavenging increases with concentration. Comparisons of each part of the plant suggest that the leaves resulted in slightly higher antioxidant properties compared to the core, for all combinations of red/white cabbage, fermentation time and concentration.

When comparing white and red cabbage then for the core one can observe higher values denoted for red cabbage in all concentrations used, while a comparison for the leaves shows similar values in lower concentrations and better results for red cabbage when observed in higher concentrations.

The effect of fermentation was mostly positive; measurements increased with fermentation time for each combination of white/red cabbage, plant part and concentration, except for the lowest concentration where rather fluctuating behavior was observed.

**Figure 3:**
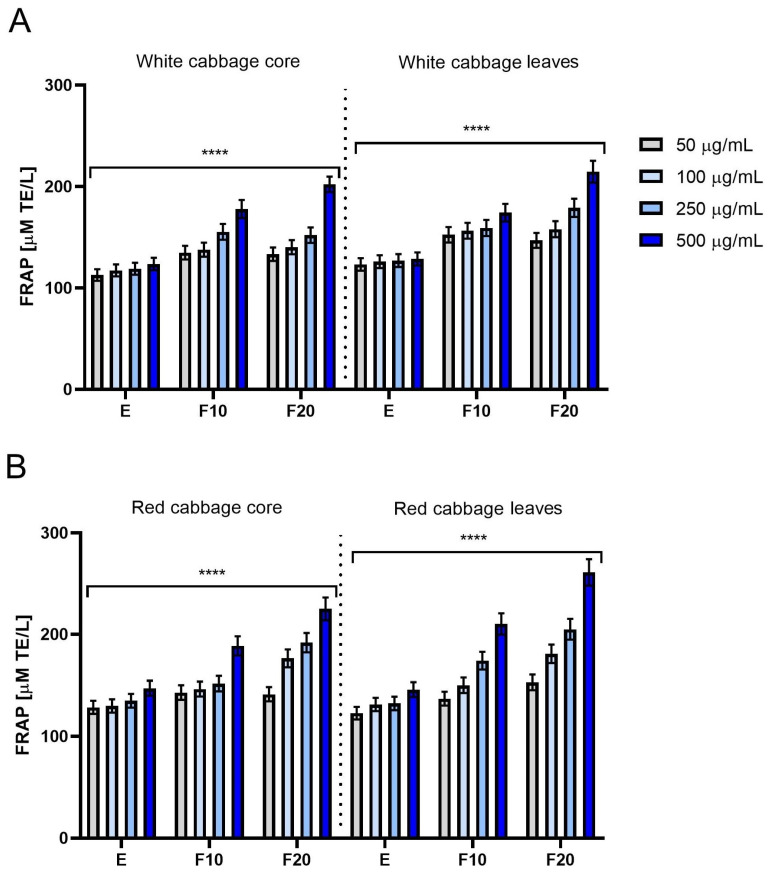
The ability of extracts (E) and ferments (F10 and F20) obtained from white cabbage core and leaves (**A**) and from red cabbage core and leaves (**B**) to reduce iron ions at concentrations of 50, 100, 250 and 500 µg/mL. The data are presented as mean ± SD from three independent experiments, with each sample tested in triplicate **** *p* < 0.0001.

The most promising results among all the samples were obtained for red cabbage leaf, with F20 ferments in the highest concentration (Figure 3).

Although similar overall trends were observed in the DPPH, ABTS, and FRAP assays, some differences between the methods were noticeable, particularly at lower concentrations and when comparing leaf and core samples. These differences are expected, since each assay is based on a different reaction mechanism and may respond differently to specific groups of antioxidant compounds. The ABTS assay is considered more sensitive toward both hydrophilic and lipophilic antioxidants, whereas DPPH is more strongly associated with the scavenging activity of compounds soluble in organic systems. In contrast, the FRAP assay reflects reducing power rather than direct radical scavenging ability. Therefore, variations observed between assays may indicate differences in the composition and reactivity of metabolites present in the extracts and ferments.

The higher antioxidant activity observed for the red cabbage samples is most likely associated with the presence of anthocyanins, which are known to exhibit strong radical scavenging and reducing properties [46]. Moreover, the increase in antioxidant activity after fermentation may result from the biotransformation of phytochemicals by microorganisms present in the SCOBY. Fermentation can lead to the release of bound phenolic compounds, formation of low-molecular-weight metabolites, and improved bioavailability of antioxidant compounds, which together may enhance the antioxidant potential of the samples [47]. The different behavior observed at lower concentrations may also suggest that the antioxidant activity of the analyzed samples depends not only on the total content of antioxidant compounds, but also on their qualitative composition and possible synergistic interactions between metabolites [45,48]. The results indicate that the leaves and core of white and red cabbage contain compounds with antioxidant potential. In the vast majority of analyses, extracts and, especially, kombucha ferments obtained from the leaves and core of white and red cabbage demonstrated higher antioxidant potential compared to unfermented material. This is consistent with the literature data indicating that kombucha fermentation of many plant materials increases antioxidant potential, an effect associated with the bioavailability of components and the release of phenolic compounds. Furthermore, SCOBY fermentation leads to a decrease in pH, which may contribute to the stability of some compounds with antioxidant potential. In the case of red cabbage, it should also be noted that the acidic fermentation environment promotes the stabilization of some pigment compounds [49,50,51]. The chromatographic results obtained in this study confirm the antioxidant potential previously reported by other researchers [8,52]. During the study, phenolic compounds such as phenolic acids and their esters with quinic acid, as well as numerous glycoside flavonoids, were detected, characterized by high antioxidant activity. Among the non-flavonoid compounds, compounds such as gallic acid, galloylquinic acids, and chlorogenic acids were identified, which can significantly increase antioxidant potential (Table 1 and Table 2).

Salek et al. [53] reached similar conclusions regarding the effect of the fermentation process on antioxidant potential. In the study, sauerkraut juice was fermented with symbiotic cultures such as kombucha. An increase in antioxidant potential during fermentation was demonstrated, as was the effect of fermentation time and starter type on the content of bioactive compounds. At the same time, the literature on classical lactic acid fermentation of cabbage indicates that this process can also significantly enhance the antioxidant potential of raw materials. Kusznierewicz et al. [54] reported a three-fold increase in activity in the ABTS test for sauerkraut juice after 14 days of fermentation. Ciska et al. [55], on the other hand, confirmed the significant antioxidant activity of fermented extracts, linking it to changes in the pool of phenolic compounds occurring during fermentation. In summary, fermentation with kombucha culture, compared to lactic acid fermentation, involves a different consortium of microorganisms, which allows for additional modification of the profile of bioactive compound metabolism and final antioxidant activity.

#### 2.2.2. Intracellular ROS Levels in Skin Cells

In the next stage of the study, the antioxidant potential of the tested extracts and ferments was assessed, analyzing their effect on the level of reactive oxygen species (ROS) in skin cells. Excessive ROS generation leads to oxidative stress, causing oxidative damage to lipids, proteins, and DNA, and consequently disrupting cellular homeostasis and tissue integrity. This negatively impacts skin function, promoting inflammatory processes and accelerating aging [56,57]. ROS determinations were performed in HaCaT keratinocytes and BJ fibroblasts using the dye H_2_DCFDA, which is oxidized to fluorescent DCF in the presence of ROS, enabling quantitative assessment of oxidative stress. To ensure proper interpretation of the intracellular ROS assay results, the kombucha starter solution alone was also evaluated in parallel to determine its potential effect on ROS generation in the tested cell lines (Figure S5).

**Figure 4:**
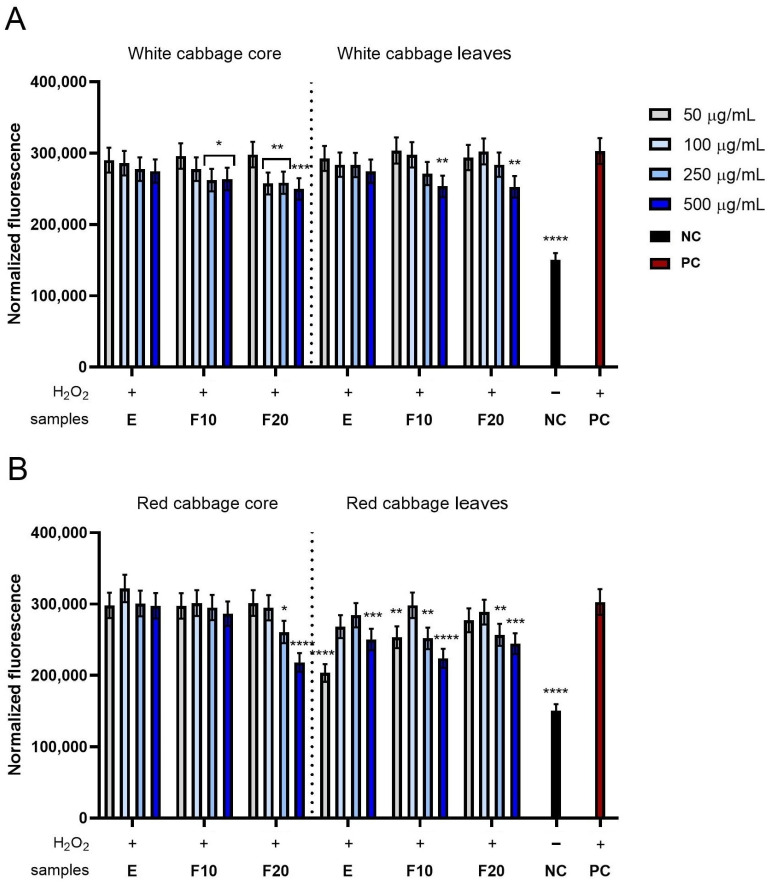
The ability of extracts (E) and ferments (F10 and F20) obtained from white cabbage core and leaves (**A**) and from red cabbage core and leaves (**B**) on the intracellular level of reactive oxygen species in fibroblasts (HDFs), at concentrations of 50, 100, 250 and 500 µg/mL. Cells cultured in a medium without the tested extracts served as the negative control (NC), while cells stimulated with hydrogen peroxide (H_2_O_2_) were used as the positive control (PC). The data are presented as mean ± SD from three independent experiments, with each sample tested in triplicate **** *p* < 0.0001, *** *p* < 0.001, ** *p* < 0.01, * *p* < 0.05.

Analysis of normalized fluorescence intensity in HDF cells used for intracellular ROS determination showed that all tested samples reduced ROS levels below those observed for the positive control, particularly at higher concentrations, with a noticeable beneficial effect of fermentation (Figure 4). For white cabbage, the difference between the core and leaves is visible only in case of F20 ferments as the results obtained for the extracts are very close; F10 ferments are also similar, while for F20 ferments the core provided lower values in high and moderate concentrations compared to the leaves. In summary, white cabbage core seems to perform only slightly better than the leaves, but only in the case of the F20 ferment (Figure 4A). For red cabbage, the comparison between the core and leaves provided more visible differences. The extracts and F10 ferments from red cabbage gave values close to the positive control for all the concentrations, while for the extract and F10 ferments from the leaves, the values were significantly lower than the positive control in most concentrations. The comparison between the core and leaves for the F20 ferments from red cabbage shows low values for both (Figure 4B).

When analyzing the effect of fermentation it is apparent that in most variants fermentation resulted in lowered normalized fluorescence, which is most noticeable between the F20 ferments compared to the extracts in higher concentrations.

Among the ferments, in the case of white cabbage the best result were observed for the F20 ferment from the core in the highest concentration of 500 µg/mL and only slightly weaker results for the F10 and F20 ferments from white cabbage leaves in the same highest concentration. All those results remain statistically below the positive control. In the case of the ferments from red cabbage, the best results were observed for the F20 ferment from the core and the F10 ferment from the leaves, both in the highest concentration. It is worth noting that the result obtained for the F20 ferment from the red cabbage core was the most promising among all the ferments, with strong statistical difference from the positive control (Figure 4).

**Figure 5:**
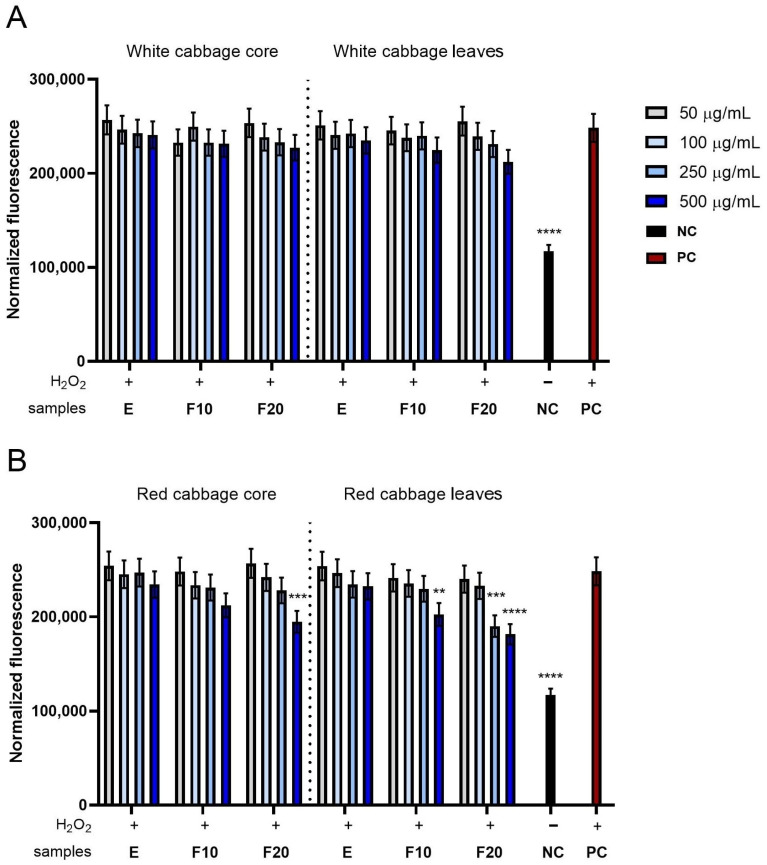
The ability of extracts (E) and ferments (F10 and F20) obtained from white cabbage core and leaves (**A**) and from red cabbage core and leaves (**B**) on the intracellular level of reactive oxygen species in keratinocytes (HaCaT), at concentrations of 50, 100, 250 and 500 µg/mL. Cells cultured in a medium without the tested extracts served as the negative control (NC), while cells stimulated with hydrogen peroxide (H_2_O_2_) were used as the positive control (PC). The data are presented as mean ± SD from three independent experiments, with each sample tested in triplicate **** *p* < 0.0001, *** *p* = 0.0001, ** *p* < 0.0056.

Analysis of normalized fluorescence intensity in the HaCaT keratinocytes used for intracellular ROS determination revealed that most of the tested samples reduced ROS levels below those observed for the positive control, whereas at the lowest tested concentrations slightly higher fluorescence values were occasionally detected. In moderate and higher concentrations though, the results remain below the positive control. It is apparent that for all the cases, increased concentration results in lower normalized fluorescence. Also, there is a visible preferable effect of fermentation, especially in higher concentrations (Figure 5).

For white cabbage, difference between the core and leaves is visible only in the case of the F10 and F20 ferments in the highest concentration. In those cases, the values registered for the leaves are slightly lower than for the core, but still not significantly lower compared to the positive control. All other measurements remain very close between the core and leaves (Figure 5A).

For red cabbage, comparison between the core and leaves provided more visible differences. The measurements collected for extracts remain very close or the same. Differences begin to be visible for the ferments. For the F10 ferments, the highest concentration value for the leaves is lower than for the core, and is also significantly lower than the positive control. For the F20 ferments the difference is even more visible, as the results for all the concentrations are lower for the leaves compared to the core (Figure 5B).

The fermentation process produces preferable effect for both white and red cabbage, but in the case of red cabbage the advantage is stronger. In the case of white cabbage a slight drop of normalized fluorescence, especially in higher concentrations, is visible. In the case of red cabbage a drop is far more significant as values recorded after fermentation in higher concentrations drop below the positive control in a statistically significant way.

Among the ferments, in the case of white cabbage the best result was observed for the F20 ferment from white cabbage leaves in the highest concentration of 500 µg/mL, but still remaining not statistically different than the positive control. In the case of the ferments from red cabbage, the best results were observed for the F20 ferment from the leaves, and only a slightly worse result was observed for the F20 ferment from the core, both in the highest concentration. It is worth noting that the result obtained for the F20 ferment from red cabbage leaves was the most promising among all the ferments, with strong statistical difference from the positive control (Figure 5).

Evaluating the level of reactive forms of ROS detection in keratinocytes and fibroblasts provides important results regarding antioxidant activity, using methods such as ABTS, DPPH, and FRAP. These tests assess the ability to neutralize radicals or reduce metal ions in cell-free systems. Determining the antioxidant potential of ROS in a cellular model allows for the development of oxidative potential, which translates into the biological protection of cells against oxidative stress. This is particularly important for keratinocytes and fibroblasts, which are at risk due to pathogenic factors that induce oxidative stress. Excessive ROS production may lead to damage to lipids, proteins, and DNA, as well as activation of pro-inflammatory pathways and accelerated skin aging [58,59]. Our research results on ROS analysis in keratinocytes and fibroblasts reveal that cabbage-derived extracts and ferments can modulate the cellular redox response. The significance of the results obtained for the fermented preparations stems from the fact that kombucha fermentation can not only increase antioxidant potential in informative assays but also limit intracellular ROS accumulation. This effect may be due to the presence of phenolics, organic phenolic transformation products, and, in the case of red cabbage, anthocyanins. These compounds may act directly, by neutralizing reactive species, or indirectly, by modulating the retail pathways of the antioxidant response and the functional pathway. 

The protective mechanism of cabbage ferments may result from several overlapping mechanisms. First, fermentation of the kombucha consortium species may be limited to the hydrolysis of plant structures and the release of phenolic substances from their bound form, which increases their bioavailability. Second, the microorganisms present in SCOBYs may contain metabolites of greater biodiversity, including free phenolic acids and other low-molecular-weight metabolites. Third, acids produced by fermentation can be included in selected bioactive products. This document highlights the importance of an acidic fermentation environment, which stabilizes anthocyanins and provides antioxidant properties [10,60].

These observations are consistent with the literature data available for fermented plant extracts. Ziemlewska et al. demonstrated that kombucha ferments are produced from fruits rich in polyphenols, which may have an effect on skin cells exposed to H_2_O_2_-induced oxidative stress [60]. Research on other plant ferments indicates that the fermentation process can enhance antioxidant activity by increasing the content of phenolic substances, polysaccharides, peptides, and metabolites formed as a result of microbial hydrolysis and biotransformation [10]. ROS analysis indicates that fermentation occurs in cabbage, which may have cytoprotective activity against keratinocytes and fibroblasts by limiting intracellular oxidative stress. These results complement the data obtained from the ABTS, DPPH, and FRAP assays, indicating that the antioxidant activity of ferments is important also in the cellular model.

### 2.3. Cytotoxicity Assessment

The next stage of the study assessed the cytotoxic properties of extracts and ferments obtained from white and red cabbage. Analyses were conducted on both the leaves and core of both cabbages at concentrations of 50, 100, 250 and 500 µg/mL. Two tests were used to assess the effect of the extracts and ferments on skin cells, Alamar Blue (AB) and Neutral Red (NR), allowing for a broad characterization of the cellular response. The AB test allows for the assessment of cellular metabolic activity by measuring the ability of mitochondria to reduce resazurin. The NR test, on the other hand, allowed for the assessment of lysosomal integrity and functionality through the accumulation of the dye in living cells. The combination of these two tests allows for the distinction between changes in cellular metabolism and damage to intracellular structures and is used in cytotoxicity and cytoprotection studies in fibroblast (HDF) and keratinocyte (HaCaT) models. To ensure accurate interpretation of the cytotoxicity results, the kombucha starter solution alone was additionally tested in parallel in order to evaluate its potential effect on the viability of the examined cell lines (Figure S6).

In the Alamar Blue Assay on HDFs, for the red cabbage overall higher values were observed for the core, while for white cabbage higher values were observed for the leaves. For the extract and F10 ferment the highest cell viability values were observed for the lowest concentration and they clearly decreased with increased concentration. For the F20 ferment, though, the highest value of cell viability was reached for the moderate concentration of 100 µg/mL, decreasing after exceeding that value.

In the experiment with red cabbage, for the extract and F10 ferment from the core, and for the extract from the leaves, the highest values of cell viability were observed for the lowest concentration and they decreased with increased concentration. In other cases such as the F20 ferment from the core and the F10 and F20 ferments from the leaves, applying a moderate concentration of 100 µg/mL resulted in the highest observed cell viability, and it decreased for higher concentrations.

In the white cabbage leaf a positive result of fermentation was observed, as the highest values of cell viability were observed for two concentrations for the 20-day ferments (50 and 100 µg/mL) compared to one concentration in the case of the extract (only 50 µg/mL). This suggests that applying the fermentation process allows for using higher concentrations with no negative effect on cell viability, as is observed in the case of the extracts. Similarly in the case of the white cabbage core, applying the fermentation process makes it possible to use the increased concentration of 100 µg/mL (F20) instead of 50 µg/mL (ferment) and keep cell viability on a similar level.

**Figure 6:**
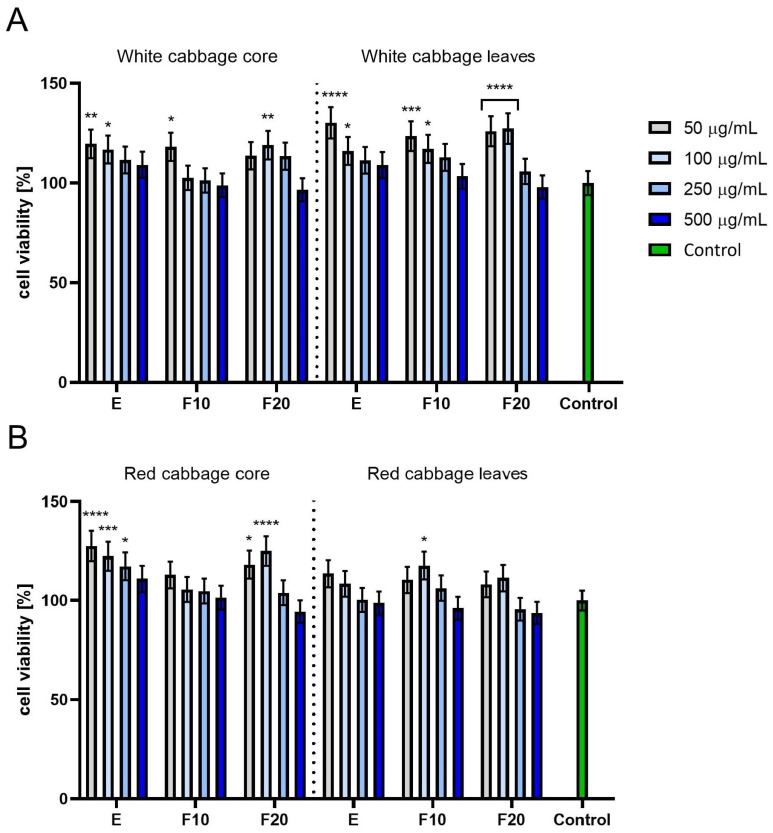
Effect of the increase in the concentration of extracts (E) and ferments (F10 and F20) obtained from white cabbage core and leaves (**A**) and from red cabbage core and leaves (**B**) (50–500 μg/mL) on cell viability (Alamar Blue assay) by cultured fibroblasts. The data are the mean ± SD of three independent experiments each consisting of three replicates per test group. **** *p* < 0.0001, *** *p* < 0.001, ** *p* < 0.01, * *p* < 0.05.

A similar conclusion can be drawn for red cabbage—applying the fermentation process allows for using increased concentrations of 100 µg/mL and keeping cell viability on the same level as in the case of the extracts in the concentration of 50 µg/mL. What is worth emphasizing is that in all of examined cases cell viability remains higher than the control, at 100% or close to that value. This means no cytotoxic effect was observed (Figure 6).

In Alamar Blue Assay on HaCaTs, for red cabbage overall higher values were observed for the core, while for white cabbage higher values were observed for the leaves. For the white cabbage core, for all variants—extract, F10 and F20 ferment—the highest values of cell viability were observed for 100 µg/mL. The highest value observed for the F20 ferment was higher than for the extract, suggesting a positive result of fermentation.

For white cabbage leaves a drop in cell viability with increased concentration was denoted. On the other hand, despite that, the absolute numbers recorded for white cabbage leaves for the concentrations of 50 and 100 µg/mL were all higher compared to the results for white cabbage core, even in its best variant, meaning F20 in the concentration of 100 µg/mL. For red cabbage core the highest values of cell viability were observed for the concentrations of 100 and 250 µg/mL (for the extract) and 100 µg/mL (for the F20 ferment). The absolute values in this case were as high as the best results for white cabbage leaves. What is more, in the case of the F20 ferments the value observed for the concentration of 100 µg/mL increased statistically significantly compared to the highest values obtained for the extract, suggesting that the fermentation process positively affected the cell viability aspect.

For red cabbage leaf, dependency on the concentration suggests that the highest value of cell viability in the case of the extract is around 250 µg/mL; in the case of the F10 ferment it is equal for 100 and 250 µg/mL and for F20 ferment it is 50 µg/mL. What is more, values for the ferments are worse than for the extract. This means that the fermentation process is rather un-preferrable in this case, resulting in decreasing overall cell viability.

**Figure 7:**
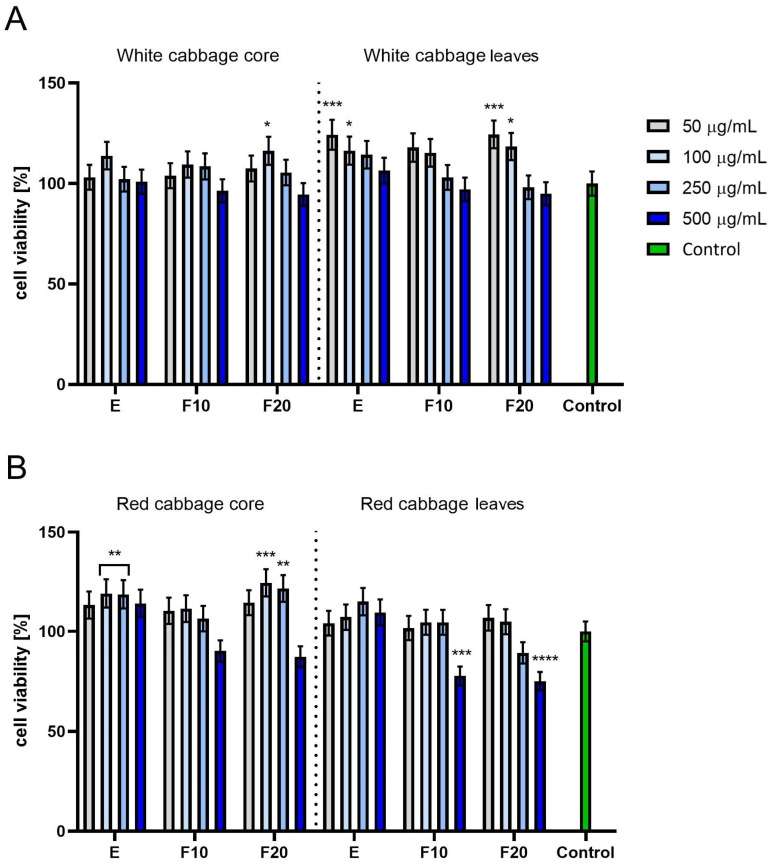
The effect of the increase in the concentration of extracts (E) and ferments (F10 and F20) obtained from white cabbage core and leaves (**A**) and from red cabbage core and leaves (**B**) (50–500 μg/mL) on cell viability (Alamar Blue assay) by cultured keratinocytes. The data are the mean ± SD of three independent experiments each consisting of three replicates per test group **** *p* < 0.0001, *** *p* < 0.001, ** *p* < 0.01, * *p* < 0.05.

What is worth emphasizing is that in most of the examined cases, cell viability remains higher than the control of 100%, with few exceptions observed for the highest concentrations when it drops to a level of approx. 80%. This means no cytotoxic effect was observed (Figure 7).

In the Neutral Red Uptake Assay on HDFs with white cabbage, the overall values observed for the leaves were higher compared to the ones observed for the core, where values for the core were not significantly higher than the control, remaining on a level close to 100% or slightly lower. In the case of white cabbage leaves, the values denoted for the extract in the lowest concentration of 50 µg/mL and the F20 ferment in the concentration of 100 µg/mL were on the same level, remaining significantly higher than the control. What is worth noting is that the highest value of cell viability for the extract was observed for the concentration of 50 µg/mL, while for the F20 ferment the same or a very close value was observed for the concentration of 100 µg/mL. This suggests that the fermentation process allows the use of a higher concentration with no drop in cell viability.

**Figure 8:**
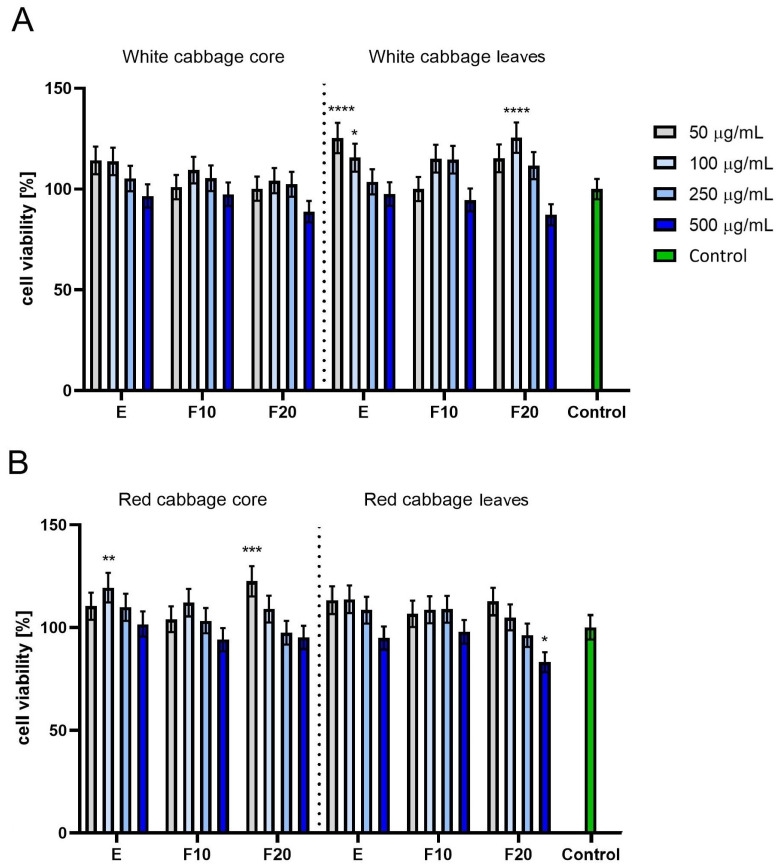
The effect of the increase in the concentration of extracts (E) and ferments (F10 and F20) obtained from white cabbage core and leaves (**A**) and from red cabbage core and leaves (**B**) (50–500 μg/mL) on cell viability (Neutral Red assay) by cultured fibroblasts. The data are the mean ± SD of three independent experiments each consisting of three replicates per test group **** *p* < 0.0001, *** *p* = 0.0004, ** *p* = 0.0041, * *p* = 0.0191.

In the experiment with red cabbage, the overall values were higher for the core than for the leaves, while the values for the leaves were not significantly higher than the control, remaining on a level close to 100% or slightly lower. For the red cabbage core, the best values of cell viability were observed for the extract in the concentration of 100 µg/mL and for the F20 ferment in the concentration of 50 µg/mL, where the value for the ferment was significantly higher, but as mentioned achieved in a lower concentration compared to the best value observed for the extract. For the red cabbage leaves, no positive effect of fermentation was observed, as a drop in cell viability was observed for the lower concentrations for the ferments compared to the extracts, plus the observed values of cell viability for the ferments were similar to the ones for the extract or even lower (Figure 8).

In the Neutral Red Uptake Assay on HaCaTs results, the overall values observed for the leaves were higher compared to the ones observed for the core, where the values for the core were not significantly higher than the control, remaining on a level close to 100% or slightly lower. For the white cabbage leaves a drop in cell viability with increased concentration was denoted. The highest values of cell viability were observed for the extract in the concentrations of 50 and 100 µg/mL and for the F20 ferment in the same concentrations, but the value for F20 in the lower concentration was significantly higher than the ones for the extract. This suggests a positive effect of fermentation.

**Figure 9:**
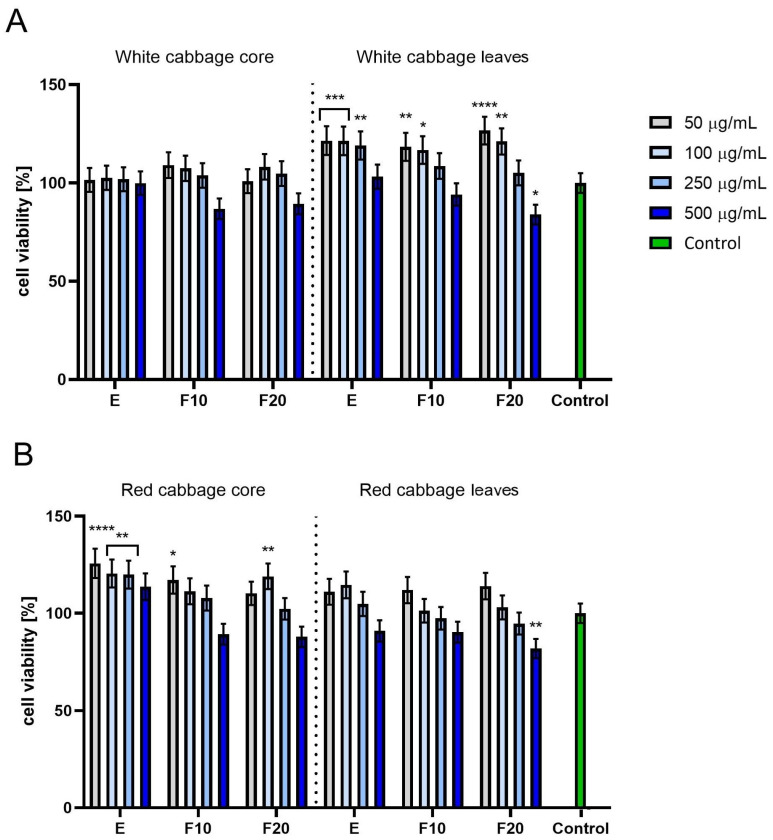
The effect of the increase in the concentration of extracts (E) and ferments (F10 and F20) obtained from white cabbage core and leaves (**A**) and from red cabbage core and leaves (**B**) (50–500 μg/mL) on cell viability (Neutral Red assay) by cultured keratinocytes. The data are the mean ± SD of three independent experiments each consisting of three replicates per test group **** *p* < 0.0001, *** *p* < 0.001, ** *p* < 0.01, * *p* < 0.05.

For red cabbage the overall values observed for the core were higher compared to the ones observed for the leaves. For the red cabbage core the highest values of cell viability were observed for the extract in the lowest concentration of 50 µg/mL, and only a bit lower were the results for the extract in 100 and 250 µg/mL. For the F20 ferment, the best values were observed for 100 µg/mL, but they were significantly lower compared to the values observed for the extract. For the red cabbage leaves, none of the recorded values was significantly higher than the control, and the ones for higher concentrations were on a level of approx. 80% only. What is more, applying fermentation resulted in cell viability dropping in lower concentrations compared to the extract (Figure 9).

The significant increase in biological activity observed after fermentation, particularly in F10 and F20, indicates that kombucha fermentation may increase the bioavailability and bioactivity of compounds present in the plant material. This effect may be related to the enzymatic degradation of cellular structures, the release of phenolic compounds, the formation of organic acids, and the biotransformation of secondary metabolites. The literature data indicate that the chemical composition of kombucha depends on the type of substrate used, fermentation time, and the microorganisms present in the inoculum. The main bioactive components of these ferments include organic acids, polyphenols, amino acids, and vitamins [60,61]. Ziemlewska et al. also demonstrated that kombucha fermentation time significantly affects the biological properties of plant ferments, including their antioxidant activity and safety against skin cells [60]. In the case of cabbage ferments, the transformation of glucosinolates and anthocyanins, which are characteristic bioactive components of Brassica plants, especially red cabbage, may be particularly important. The metabolites formed during fermentation, such as free phenolic acids, organic acids, and glucosinolate transformation products, may act as multi-directional modulators of cellular pathways essential for skin function. This may give fermented cabbage preparations a specific profile of activity against fibroblasts and keratinocytes.

At the same time, the lack of cytotoxicity of the tested preparations confirms their favorable biological safety profile. These observations are consistent with the results of You et al., who demonstrated that exosome-like nanovesicules isolated from white and red cabbage did not induce cytotoxicity against HaCaT and HDF cells and also demonstrated cell proliferation.

The conducted studies show that the samples from white cabbage leaves generally exhibited higher biological activity than the samples from red cabbage leaves. However, red cabbage cores, often treated as technological waste, demonstrated very beneficial cytoprotective effects. This highlights the potential for utilizing food industry by-products and is consistent with the concept of sustainable development. The obtained results therefore indicate that kombucha fermentation can be an effective method for increasing the health-promoting value of plant materials and their processing by-products [49,62,63,64]. Fermented cabbage preparations, thanks to their combination of beneficial biological activity and lack of cytotoxicity to skin cells, may be a promising raw material for designing functional foods or preparations with dermoprotective potential.

### 2.4. Assessment of Anti-Inflammatory Activity

In the next stage of the study, the effect of extracts and kombucha ferments obtained from white and red cabbage core and leaves on the activity of pro-inflammatory cytokines in THP-1 monocyte cells was assessed. Interleukin 1β (IL-1β) and interleukin 6 (IL-6) were selected for analysis because they are key mediators of the early inflammatory response induced by lipopolysaccharide (LPS) in monocyte–macrophage system cells. IL-1β plays an important role in the initiation and amplification of the inflammatory cascade, while IL-6 participates in both the regulation of the acute phase of inflammation and the modulation of the immune response. The assessment of the levels of these two markers allows for the analysis of the anti-inflammatory potential of the tested samples in an in vitro model. To ensure proper interpretation of the anti-inflammatory activity results, the kombucha starter solution alone was also evaluated in parallel to determine its potential influence on cytokine production in the THP-1 cell model (Figure S7).

THP-1 cells incubated with the tested extracts and ferments at concentrations of 100 and 250 µg/mL were stimulated with bacterial lipopolysaccharide (LPS) at a concentration of 10 µg/mL per well. The results obtained were presented as the fold of the activity of the tested cytokines compared to the negative control (NC), which consisted of cells not exposed to either the tested compounds or LPS. The positive control (PC) consisted of cells stimulated with LPS but without the addition of E, F10 and F20, which allowed the maximum inflammatory response to be determined. A solution of diclofenac at a concentration of 10 µg/mL per well was used as a reference control with known anti-inflammatory activity.

**Figure 10:**
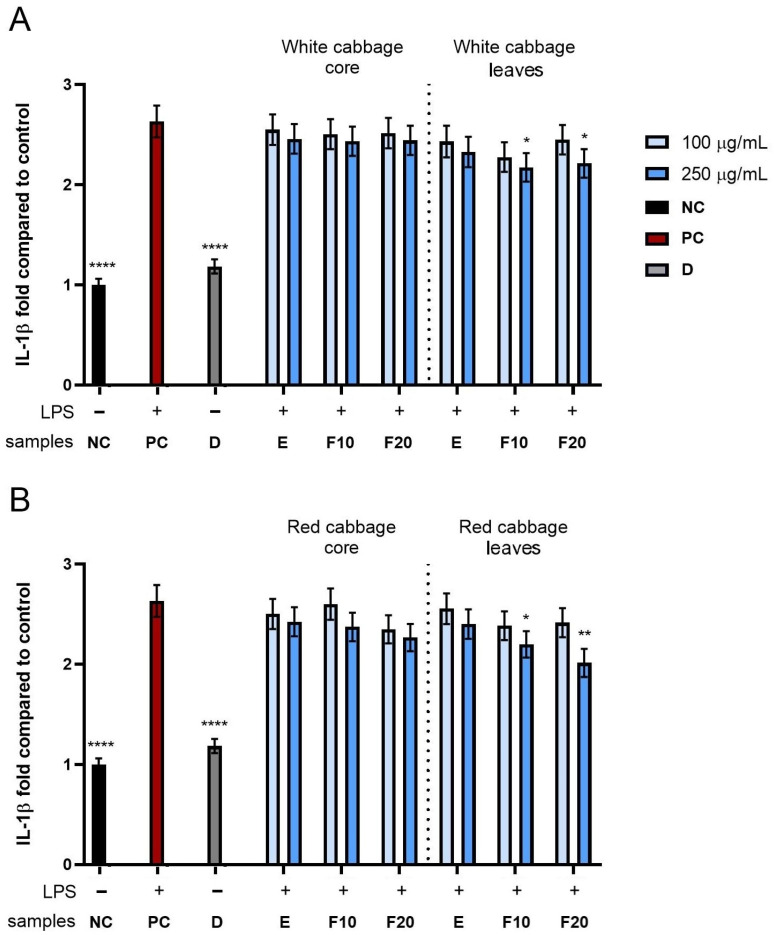
The impact of extracts (E) and ferments (F10 and F20) obtained from white cabbage core and leaves (**A**) and from red cabbage core and leaves (**B**) on IL-1β levels in THP-1 cells stimulated with bacterial LPS (10 μg/mL), expressed as fold compared to the negative control (NC). The positive control (PC) consisted of cells stimulated with LPS but without the addition of E, F10 and F20. Diclofenac (D; 10 µg/mL) was used as the reference compound. The data represent the mean ± SD of three independent experiments, with each sample tested in duplicate. **** *p* < 0.0001, ** *p* = 0.0010, * *p* < 0.05.

As shown in Figure 10, all tested compounds at the concentrations used (100 and 250 µg/mL) showed the ability to reduce IL-1β levels compared to the positive control, indicating their potential anti-inflammatory activity. The most pronounced effect was observed for ferments obtained after 10 and 20 days of fermentation from both white and red cabbage leaves, especially at a concentration of 250 µg/mL. In the case of white cabbage (Figure 10A), ferments F10 and F20 reached values of 2.17 ± 0.14 and 2.21 ± 0.15 fold compared to NC, respectively, while for red cabbage (Figure 10B), these values were 2.19 ± 0.13 and 2.01 ± 0.14 for F10 and F20, respectively.

**Figure 11:**
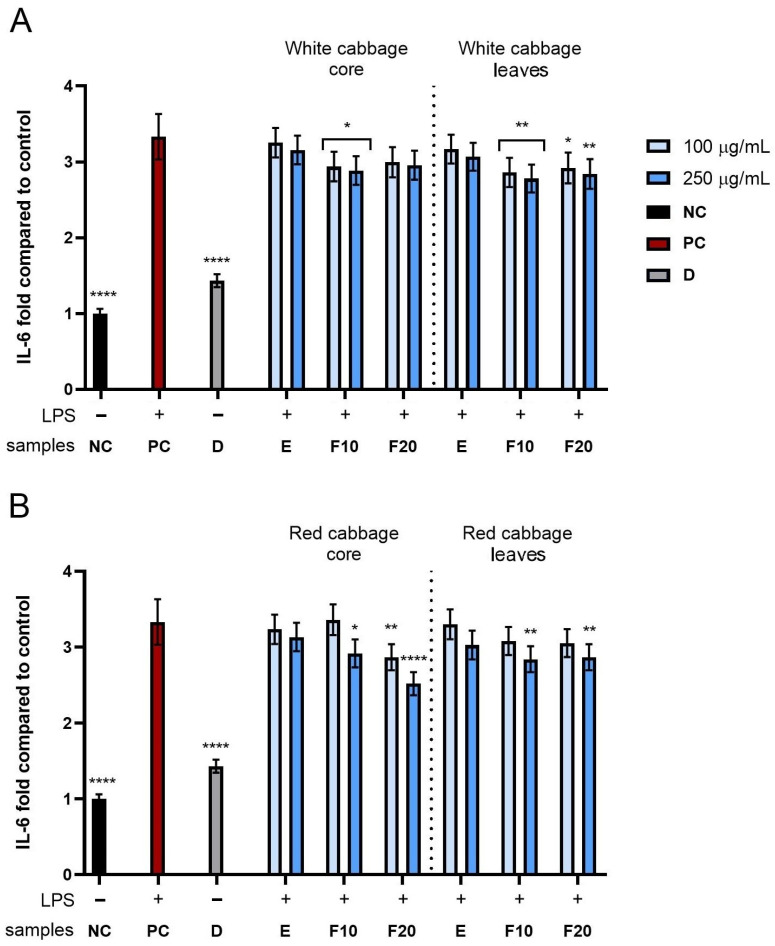
The impact of extracts (E) and ferments (F10 and F20) obtained from white cabbage core and leaves (**A**) and from red cabbage core and leaves (**B**) on IL-6 levels in THP-1 cells stimulated with bacterial LPS (10 μg/mL), expressed as fold compared to the negative control (NC). The positive control (PC) consisted of cells stimulated with LPS but without the addition of E, F10 and F20. Diclofenac (D; 10 µg/mL) was used as the reference compound. The data represent the mean ± SD of three independent experiments, with each sample tested in duplicate. **** *p* < 0.0001, ** *p* < 0.01, * *p* < 0.05.

Analysis of the effect of the tested samples on IL-6 levels (Figure 11) showed that both the extracts and ferments from the leaves and core of both cabbage varieties reduced the activity of this cytokine in the THP-1 cell model. In the case of white cabbage (Figure 11A), the kombucha ferments had a stronger anti-inflammatory effect than the corresponding extracts in both concentrations analyzed. The most favorable values were recorded for the core F10 obtaining 2.93 ± 0.20 and 2.88 ± 0.19 fold compared to the control for concentrations of 100 and 250 µg/mL, respectively, while ferments F10 and F20 achieved values of 2.78 ± 0.18 and 2.84 ± 0.17 fold at a concentration of 250 µg/mL, respectively.

A similar relationship was observed for red cabbage (Figure 11B), where the core and leaf ferments F10 and F20 showed a statistically significant reduction in IL-6 levels, especially at a higher concentration used. The strongest anti-inflammatory effect was observed for the core F20 at a concentration of 250 µg/mL, which reduced IL-6 levels to 2.52 ± 0.15-fold compared to the negative control. These results confirm that the fermentation process can significantly enhance the biological properties of plant material, increasing its potential to modulate the inflammatory response in immune system cells. The stronger anti-inflammatory effect observed in the fermented samples compared to the unfermented extracts may result not only from the increased concentration of phenolic compounds, but also from qualitative changes in their composition that occur during fermentation. As demonstrated, kombucha fermentation promotes the biotransformation of complex polyphenols into metabolites with lower molecular weights, characterized by greater bioavailability and biological activity. In particular, the enzymatic activity of microorganisms may lead to the release of bound phenolic acids and flavonoid aglycones, which can interact more effectively with intracellular inflammatory signaling pathways [47,65]. Furthermore, fermentation may contribute to the production of additional metabolites, including organic acids and glucuronic acid derivatives, which may synergistically enhance the anti-inflammatory response. Previous studies have shown that metabolites derived from kombucha can inhibit LPS-induced activation of the NF-κB and MAPK signaling pathways, leading to a reduction in the secretion of IL-1β, IL-6, and TNF-α by immune cells [66].

Available data indicate that *Brassica oleracea* var. capitata exhibits anti-inflammatory properties by modulating key inflammatory pathways and the expression of pro-inflammatory cytokines, including IL-1β and IL-6. These effects result from the synergistic action of glucosinolates, isothiocyanates, flavonols, and phenolic compounds, which simultaneously exhibit strong antioxidant properties and the ability to regulate the immune response [67,68]. These mechanisms are mainly associated with the activation of the Nrf2 pathway and the inhibition of NF-κB, which leads to a reduction in the transcription of genes encoding inflammatory mediators [69,70].

Flavonols also play an important role, especially kaempferol, which inhibits COX-1 and COX-2 activity, limiting prostaglandin synthesis and secondarily reducing IL-6 production [71]. In turn, hydroxycinnamic acid derivatives, such as p-coumaroylquinic acid isomers and chlorogenic acids, exhibit anti-inflammatory effects by reducing the expression of TNF-α and IL-6 and activating the NRF2/HO-1 pathway [72,73]. It should also be emphasized that IL-1β and IL-6 are regulated by partially distinct signaling mechanisms. The maturation of IL-1β depends largely on inflammasome activation and caspase-1 activity, whereas IL-6 production is primarily associated with transcriptional regulation via NF-κB. Therefore, the observed differences in the degree of inhibition between these cytokines may indicate that the enzymes under study simultaneously affect multiple inflammatory pathways rather than acting through a single molecular target [74].

These mechanisms have been confirmed in experimental studies. Rokayya et al. demonstrated the anti-inflammatory and antioxidant effects of cabbage preparations in in vitro models [75], while in vivo studies indicate that compounds from the *Brassicaceae* family act mainly by modulating the NF-κB pathway and the expression of inflammatory cytokines [76,77]. In a model of skin inflammation in mice, a reduction in inflammatory cell infiltration and a decrease in pro-inflammatory cytokine levels were observed [78]. Similarly, in studies on RAW 264.7 macrophage lines, extracts from various cabbage varieties significantly reduced NO production and IL-6 and IL-1β expression in LPS-induced inflammation [79]. A special role in red cabbage is attributed to anthocyanins, which modulate the inflammatory response by lowering IL-6 levels and increasing IL-10 production [80]. Anthocyanins are characterized by high antioxidant potential and the ability to inhibit inflammatory signaling mediated by reactive oxygen species (ROS). Since oxidative stress is closely linked to LPS-induced cytokine production, the enhanced antioxidant properties of red cabbage ferments may indirectly contribute to a reduction in the secretion of IL-1β and IL-6 [74].

As demonstrated in our studies, kombucha enzymes exhibited comparable and often even stronger anti-inflammatory effects, depending on the fermentation time and concentration used. The observed effect of inhibiting the production of pro-inflammatory cytokines was most likely related to the higher content of bioactive compounds identified by chromatography, as confirmed in Table 1. These compounds, including polyphenols, flavonoids, and phenolic acid derivatives, are known to modulate key inflammatory pathways and may have been responsible for the observed biological effects of the enzymes. Despite the lack of direct reports on the fermentation of white and red cabbage using kombucha, there are studies confirming the effectiveness of cabbage fermentation using selected strains of lactic acid bacteria. A study conducted on RAW264.7 macrophages showed that cabbage fermented using a mixed culture of *Lactobacillus plantarum* and *Lactobacillus acidophilus* significantly inhibited the LPS-induced inflammatory response. This preparation, in a dose-dependent manner, reduced the production of nitric oxide (NO), prostaglandin E2 (PGE2) and pro-inflammatory cytokines IL-1β and TNF-α, without showing cytotoxicity at concentrations up to 1000 μg/mL. In addition, a reduction in the expression of iNOS and COX-2 proteins and inhibition of NF-κB nuclear translocation and IκB inhibitor degradation were observed [81]. Importantly, the anti-inflammatory effect observed in this study was achieved without any previously reported cytotoxic effects at the concentrations tested, suggesting that the reduction in cytokine levels was associated with an immunomodulatory effect rather than with reduced cell viability. This aspect is of particular importance in the context of potential applications of preparations derived from fermented extracts in cosmetics and pharmaceuticals. It should be noted, however, that this study was conducted using a single in vitro model based on LPS-stimulated THP-1 monocytes. Although this cell model is widely used to assess anti-inflammatory activity, additional studies using other immune cell models, analysis of intracellular signaling proteins, and in vivo experiments are necessary to fully elucidate the mechanisms responsible for the observed biological effects.

### 2.5. Assessment of Antibacterial Activity

The antibacterial activity of aqueous extracts (E) and kombucha-fermented preparations obtained after 10 and 20 days of fermentation (F10 and F20) from white and red cabbage (core and outer leaves) was evaluated against five bacterial strains. To the best of our knowledge, the antibacterial activity of cabbage by-products subjected to SCOBY-based fermentation has not been previously characterized using MIC-based assays. The results demonstrated a clear dependence of antimicrobial efficacy on fermentation time, plant part, cabbage type, and bacterial species (Table 3). Fermented preparations exhibited stronger antibacterial activity than the corresponding aqueous extracts. A gradual improvement in activity from the extracts to F10 and subsequently to F20 fermented extracts was observed in most cases, suggesting that SCOBY-driven fermentation modified the phytochemical composition of the samples and enhanced the availability of bioactive metabolites with antibacterial properties. Among all the tested samples, red cabbage leaf F20 displayed the strongest activity, particularly against *Staphylococcus aureus* (MIC = 154 µg/mL), followed by red cabbage leaf F10 (210 µg/mL) and white cabbage core F20 (260 µg/mL). In contrast, several aqueous extracts showed weak or no detectable inhibition (MIC > 500 µg/mL), especially against Gram-negative strains.

Gram-positive bacteria (*Staphylococcus aureus*, *Staphylococcus capitis*, and *Micrococcus luteus*) were consistently more susceptible to the tested preparations than Gram-negative strains. This pattern is in agreement with previously reported observations for polyphenol-rich plant extracts, which frequently exert stronger inhibitory effects against Gram-positive organisms [82]. The increased susceptibility of Gram-positive bacteria is commonly attributed to structural differences in the cell envelope, particularly the absence of an outer membrane that limits the penetration of bioactive compounds [83]. In contrast, *Escherichia coli* exhibited moderate sensitivity, while *Pseudomonas aeruginosa* was the most resistant microorganism tested. Only the fermented preparations showed measurable inhibitory activity against *P. aeruginosa*, and MIC values remained relatively high (≥368 µg/mL). The intrinsic resistance of this species has been widely associated with reduced outer membrane permeability and efficient efflux systems [84].

Chromatographic analysis revealed that fermentation increased the concentration of several phenolic compounds, including gallic acid, galloylquinic acids, chlorogenic acids, catechin/epicatechin, and flavonol derivatives (quercetin and kaempferol glycosides) (Table 1 and Table 2). Interestingly, samples characterized by the highest abundance of these compounds, particularly red cabbage leaf F20, also demonstrated the lowest MIC values against the tested strains. The enhanced antibacterial activity observed for fermented samples may therefore be associated with fermentation-induced modifications of the phytochemical profile of these samples, particularly the increased abundance of phenolic acids and flavonoid derivatives observed after fermentation. Phenolic acids and flavonoids have been reported to interact with bacterial membranes, alter membrane permeability, interfere with enzymatic activity, and disrupt intracellular homeostasis [85]. Catechin and epicatechin, in particular, have been described as membrane-active compounds capable of affecting cell integrity, while gallic and chlorogenic acids have been associated with growth inhibition through multi-target mechanisms [86,87,88]. The pronounced antibacterial activity observed for red cabbage leaf F20 likely results from interactions between multiple phenolic constituents and fermentation-derived metabolites, which together may enhance the overall antibacterial potential of the sample. The leaf fractions generally contained higher levels of several quantified phenolics compared with the core fractions, which may partially explain their stronger antibacterial effects. Importantly, all stock solutions were prepared in PBS and exhibited near-neutral pH values (extracts: 7.11–7.15; F10: 6.92–6.98; F20: 6.88–6.94). After dilution in Mueller–Hinton broth (pH 7.25), the assay conditions remained close to neutral. Therefore, the contribution of acidity to antibacterial activity appears limited, and the observed inhibitory effects are more likely associated with phytochemical composition and fermentation-derived metabolites rather than pH-dependent mechanisms. Although the quantified phenolic compounds likely contribute to the observed antibacterial effects, the magnitude of activity cannot be fully attributed to individual compounds alone. The antimicrobial efficacy of complex plant matrices is often enhanced by synergistic interactions among multiple constituents. Moreover, fermentation may facilitate the release of bound phenolics from the plant matrix or promote the formation of additional bioactive metabolites not included in the quantitative panel [89]. In Brassica species, the presence of glucosinolate-derived compounds and their transformation products may also contribute to biological activity [90,91]. Although these compounds were not directly quantified in the present analysis, their potential involvement cannot be excluded. Taken together, the results indicate that fermentation enhances the antibacterial activity of cabbage-derived preparations, particularly against Gram-positive bacteria, and that this effect is likely driven by qualitative and quantitative changes in phytochemical composition rather than by differences in pH.

Previous studies indicate that preparations derived from *Brassica oleracea* may exhibit antibacterial activity, most consistently against *Staphylococcus aureus* and, to a lesser extent, Gram-negative bacteria such as *Escherichia coli* [92,93]. In particular, a methanolic red cabbage extract was reported to inhibit *S. aureus* and *E. coli*, with ultrastructural observations suggesting cell envelope damage in exposed bacteria [92]. The antimicrobial efficacy of cabbage-derived preparations is strongly influenced by the chemical composition of the extracted fraction, as lipophilic fractions isolated from cabbage leaves were shown to inhibit *S. aureus* and *Pseudomonas aeruginosa* [94]. Taken together, these findings suggest that phenolic-rich red cabbage extracts contribute to antibacterial activity against both Gram-positive and Gram-negative bacteria. Fermentation has been reported to modulate the bioactivity of vegetable matrices, and diffusion-based assays demonstrated that fermented vegetable extracts, including fermented white cabbage, can inhibit *S. aureus*, *E. coli* and *P. aeruginosa* [62]. Moreover, substrate type and fermentation time are recognized as major determinants shaping the final bioactive profile of kombucha-type products [4].

In this context, the present study provides new data on cabbage-derived preparations subjected to SCOBY-driven fermentation and evaluated using a standardized microdilution MIC assay. Although previous studies have demonstrated that kombucha starters can be applied to ferment cabbage-based substrates, these investigations have mainly focused on physicochemical and microbiological changes rather than on antibacterial efficacy against defined pathogenic strains [53]. To date, the antimicrobial performance of such fermented cabbage preparations has not been thoroughly characterized using quantitative MIC-based approaches. The results obtained here indicate that kombucha fermentation may alter the biological properties of cabbage-derived preparations, enhancing their antibacterial potential compared with aqueous extracts alone.

## 3. Materials and Methods

### 3.1. Plant Material, Extraction and Fermentation Procedure

White cabbage and red cabbage were obtained from a local producer. For each sample, 18 g of core and leaf material was mixed with 600 mL of distilled water and extracted under magnetic stirring for 24 h. The resulting extracts were then sonicated for 30 min in an ultrasonic bath (Digital Ultrasonic Cleaner, Thermo Fisher Scientific, Waltham, MA, USA) at room temperature. A kombucha starter culture (SCOBY) purchased from a commercial supplier (Nasza Przyszłość, Szczecinek, Poland) was used for the fermentation process. The SCOBY used in this study consisted of a symbiotic consortium of acetic acid bacteria (AAB), *Acetobacter* and *Gluconobacter*, along with various yeast species (e.g., *Saccharomyces*, *Zygosaccharomyces*, *Candida*, *Pichia*), involved in sugar fermentation. Portions of 200 mL of each extract were transferred to sterile 1000 mL beakers and supplemented with sucrose and SCOBY to a final concentration of 10.0% (*w*/*v*). The extraction conditions were selected to improve the recovery of water-soluble compounds from the structurally rigid cabbage tissues, including the outer leaves and cores. Extraction was performed at room temperature and without additional heating to minimize degradation of temperature-sensitive metabolites. Fermentation was carried out at 25 °C, which is considered an optimal temperature for kombucha fermentation, ensuring balanced metabolic activity of yeasts and acetic acid bacteria and stable fermentation conditions [95]. The fermented samples were labeled F10 and F20, while the non-fermented extracts were denoted as E.

### 3.2. UHPLC–DAD–MS Analysis

The analytical approach applied in this study was based on our previously developed and validated UHPLC–DAD–MS procedure, with minor modifications adapted to the analyzed matrices [96]. UHPLC analyses were performed using an Infinity Series II system (Agilent Technologies, Santa Clara, CA, USA) coupled to a diode array detector and an ESI–TOF mass spectrometer (Agilent 6224). Chromatographic separation was achieved on a Kinetex C18 column (1.7 µm, 150 mm × 2.1 mm; Phenomenex, Torrence, CA, USA) maintained at 30 °C. The mobile phase consisted of water acidified with 0.05% formic acid (A) and acetonitrile containing 0.05% formic acid (B), delivered at a flow rate of 0.2 mL/min. Phenolic compounds were separated using a gradient elution from 98% A to 35% A over 85 min. Amino acids and low-molecular-weight organic acids were analyzed using a gradient starting at 100% A and decreasing to 75% A within 20 min. Mass spectrometric detection was carried out using electrospray ionization. The drying gas temperature was set to 325 °C with a flow rate of 8 L/min, the nebulizer pressure was 30 psi, and the capillary voltage was 3500 V. The skimmer voltage was 65 V, while fragmentor voltages were set to 140 and 200 V in negative ion mode for low-molecular-weight organic acid and phenolic compounds, respectively, and 120 V in positive ion mode for amino acids. The quantification of metabolites was performed based on calibration curves obtained for authentic standards. In cases where authentic reference standards were not available, quantification was carried out using the calibration curves of structurally related compounds with similar physicochemical properties.

### 3.3. Determination of Antioxidant Properties

#### 3.3.1. ABTS Scavenging Assay

To determine the antioxidant potential of white and red cabbage root and leaf extracts and ferments, the ABTS radical scavenging assay, as previously described by Miller et al. [97], was performed. A working solution was prepared by reacting 7 mM ABTS with 2.4 mM potassium persulfate (1:1 ratio) for 14 h at 22 °C, then diluted with PBS to reach an initial absorbance of 1.0 ± 0.04 at λ = 734 nm. Samples at concentrations of 50, 100, 250 and 500 µg/mL were combined with the ABTS reagent, and their absorbance was recorded using a UV/VIS spectrophotometer (Thermo Fisher Scientific, Waltham, MA, USA). Trolox and ascorbic acid served as positive standards, while distilled water was used as a negative control. The radical scavenging activity was calculated as a percentage relative to the control, based on three independent experiments performed in triplicate.

(1) $$\%\;ABTS\;scavenging=\left(1-\left(\frac{Abs\;sample}{Abs\;control}\right)\right)\times 100$$

#### 3.3.2. DPPH (1,1-Diphenyl-2-Picrylhydrazyl) Radical Scavenging Assay

To determine the antioxidant capacity of the white cabbage and red cabbage core and leaf extracts and ferments, a DPPH radical scavenging assay was performed, as described by Brand-Williams et al. [37]. Briefly, 100 µL of a 4 mM DPPH methanolic solution was added to the samples at concentrations of 50, 100, 250 and 500 µg/mL in 96-well plates. Absorbance was measured at λ = 517 nm after 20 min of incubation using a plate reader. Trolox and ascorbic acid served as positive controls, while distilled water was used as the negative control. The results, derived from three independent experiments performed in triplicate, were expressed as the percentage of DPPH radical inhibition relative to the control.

(2) $$\%\;DPPH\;scavenging=\frac{Abs\;control-Abs\;sample}{Abs\;control}\times 100$$

#### 3.3.3. Determination of Ferric Reducing Antioxidant Power (FRAP Assay)

The antioxidant potential of the samples was assessed using the FRAP (ferric reducing antioxidant power) assay, adapted from the method of Benzie and Strain [98]. The FRAP reagent was freshly prepared by mixing 0.3 M acetate buffer, TPTZ (2,4,6-tripyridyl-s-triazine), and FeCl_3_ × 6H_2_O in a 10:1:1 ratio (Merck KGaA, Darmstadt, Germany). For analysis, 180 µL of the FRAP reagent and 20 µL of each sample were combined in a 96-well plate, with distilled water used as the blank. After 20 min of incubation, absorbance was recorded at 593 nm. A calibration curve was generated using Trolox (0–800 µM). All measurements were performed in triplicate in three independent experiments, and the results were expressed as µmol Trolox equivalents per liter (µmol TE/L).

#### 3.3.4. Determination of Intracellular Levels of Reactive Oxygen Species (ROS)

To determine the ability of the white and red cabbage core and leaf extract and ferment to inhibit the intracellular production of reactive oxygen species in skin cells, the reduction in oxidative stress was evaluated in keratinocytes (HaCaT) and fibroblasts (BJ) using the fluorogenic probe H_2_DCFDA (2′,7′-dichlorodihydrofluorescein diacetate) [99]. Cells were incubated for 24 h with the tested samples prepared in DMEM at concentrations of 50, 100, 250 and 500 µg/mL. Subsequently, the medium was replaced with serum-free DMEM containing 10 µM H_2_DCFDA. Oxidative stress was induced by the addition of hydrogen peroxide (final concentration 500 µM) to the samples and positive control. After 60 min of incubation, fluorescence was measured at 485 nm (excitation) and 530 nm (emission) using a microplate reader (FilterMax F5, Thermo Fisher Scientific). All experiments were conducted in triplicate and repeated three times independently. The results were expressed as a percentage of the positive control.

### 3.4. Cytotoxicity Analysis

#### 3.4.1. Cell Culture

Cytotoxicity and intracellular ROS levels were evaluated using human dermal fibroblasts (HDFs) and keratinocytes (HaCaTs) sourced from CLS (Cell Lines Service; Eppelheim, Germany) [25]. Anti-inflammatory properties were determined using the THP-1 cell line obtained from Merck (Merck, KGaA, Darmstadt, Germany) [100]. HDFs and HaCaTs were maintained in high-glucose DMEM (dulbecco’s modified eagle medium) supplemented with 10% FBS and 1% penicillin/streptomycin (100 U/mL and 1000 µg/mL, respectively). THP-1 were maintained in RPMI1640 (Roswell Park Memorial Institute 1640; Genos, Łódź, Poland) supplemented with 10% FBS and 1% penicillin/streptomycin (100 U/mL and 1000 µg/mL, respectively). Cells were grown in a humidified incubator at 37 °C with 5% CO_2_. Upon reaching 70–80% confluence, the layers were washed with PBS, detached using trypsin, and resuspended in fresh medium. For subsequent assays, the cells were seeded into 96-well plates at a density of 1 × 10^4^ cells per well and incubated for 24 h to allow for proper attachment.

#### 3.4.2. Alamar Blue Assay

Cell viability was initially assessed using the Alamar Blue assay, adapted from the method described by Page et al. [101]. After 24 h of seeding in 96-well plates, skin cells were exposed to the tested extracts and ferments at concentrations of 50, 100, 250 and 500 µg/mL for an additional 24 h. The treatment medium was then supplemented with resazurin (final concentration 60 µM; Merck KGaA, Darmstadt, Germany). Untreated cells were used as the control. Following a 2 h incubation period, fluorescence was recorded at an emission wavelength of 570 nm using a microplate reader (Thermo Fisher Scientific). All experiments were conducted in triplicate and repeated independently three times for each tested concentration.

(3) $$cell\;viability\;[\%]=\frac{Abs\;sample}{Abs\;control}\times 100$$

#### 3.4.3. Neutral Red Uptake Assay

Cell viability was further evaluated using the Neutral Red assay, based on the method described by Borenfreund et al. [102]. Skin cells seeded in 96-well plates were exposed for 24 h to the tested extracts and ferments at concentrations of 50, 100, 250 and 500 µg/mL. The medium was then replaced with Neutral Red solution prepared in DMEM (Merck KGaA, Darmstadt, Germany), and HDF and HaCaT cells were incubated for 2 h. After dye removal, the cells were washed with PBS, and a decolorizing solution consisting of ethanol, acetic acid, and water (50:1:49, (*v*/*v*/*v*)) was added. Untreated cells served as the control. Absorbance was measured at 540 nm using a microplate reader. All experiments were performed in triplicate and repeated independently three times.

(4) $$cell\;viability\;[\%]=\frac{Abs\;sample}{Abs\;control}\times 100$$

### 3.5. Assessment of Anti-Inflammatory Activity

The anti-inflammatory effect of extracts from the core and leaves of white and red cabbage and their kombucha ferments was assessed by the quantitative determination of cytokines IL-1β and IL-6. THP-1 monocyte cells were stimulated with lipopolysaccharide (LPS, 10 µg/mL) derived from *Escherichia coli* O111:B4 for 24 h. During this period, the cells were simultaneously exposed to the test samples at concentrations of 100 and 250 µg/mL. The medium with the cells was then collected and centrifuged, the supernatant was aspirated, and 100 µL of RIPA buffer (radioimmunoprecipitation buffer) was added to it for cell lysis. The prepared samples were then analyzed by ELISA (Elabscience Biotechnology Inc., Houston, TX, USA) according to the manufacturer’s protocol described earlier by Nizioł-Łukaszewska et al. [25]. Absorbance was recorded at 450 nm using a FilterMax F5 microplate reader (Thermo Fisher Scientific, USA). Cells not treated with the substance served as a negative control (NC), while cells stimulated with LPS alone served as a positive control (PC). A solution of diclofenac at a concentration of 10 µg/mL per well was used as a reference control. All experiments were conducted in triplicate and repeated independently three times for each tested concentration.

### 3.6. Determination of Minimal Inhibitory Concentration (MIC) and Bacterial Viability

The antibacterial activity of aqueous extracts and kombucha-fermented preparations (F10 and F20) obtained from white and red cabbage (core and outer leaves) was determined using a resazurin-based broth microdilution method adapted from previously described protocols by Sarker et al. and Kowalska-Krochmal and Dudek-Wicher, with minor modifications [103,104]. Five bacterial strains were tested: *Staphylococcus aureus* ATCC BAA-2312™, *Staphylococcus capitis* ATCC^®^ 146™, *Micrococcus luteus* ATCC^®^ 10240™, *Escherichia coli* ATCC^®^ 25922, and *Pseudomonas aeruginosa* ATCC^®^ 35032. The strains were maintained on tryptic soy agar (TSA, Argenta, Poznan, Poland) at 37 ± 1 °C for 18–24 h prior to experiments. Fresh colonies were suspended in Mueller–Hinton broth (MHB, Argenta, Poznan, Poland) and adjusted spectrophotometrically to an optical density at 625 nm (OD_625_) of 0.080–0.100. The suspension was subsequently diluted in MHB to obtain a final inoculum of approximately 1.5 × 10^5^ CFU/mL in each well. Antibacterial activity was assessed in sterile 96-well microplates (Googlab Scientific, Rokocin, Poland). Briefly, 100 µL of stock solution (1 mg/mL) of each tested sample was added to the first row, and 100 µL of MHB was added to all remaining wells. Two-fold serial dilutions were performed to obtain final concentrations ranging from 500 to 3.9 µg/mL. Subsequently, 25 µL of the standardized bacterial suspension was added to each well. The plates were incubated at 37 °C for 20 h under aerobic conditions. Following incubation, 50 µL of 0.05% (*w*/*v*) aqueous resazurin solution was added to each well, and the plates were further incubated for 30 min at 37 °C in the dark. Fluorescence intensity was measured using a microplate reader (BioTek Synergy, Agilent Technologies, Santa Clara, CA, USA) at excitation and emission wavelengths of 530 nm and 595 nm, respectively. Appropriate controls were included in each experiment: growth control (bacteria in MHB without test samples), sterility control (MHB only), dye control (MHB with resazurin), and sample blanks (test sample with MHB and resazurin without bacteria) to account for intrinsic fluorescence and potential chemical reduction in resazurin by plant-derived matrices. Ciprofloxacin was used as a positive control for Gram-negative strains (*E. coli* and *P. aeruginosa*), whereas gentamicin was used for Gram-positive strains (*S. aureus*, *S. capitis*, and *M. luteus*). All stock solutions were prepared in PBS and were near-neutral (extracts: pH 7.11–7.15; F10: pH 6.92–6.98; F20: pH 6.88–6.94), suggesting a limited contribution of acidity to antibacterial effects. MIC was defined as the lowest concentration of the tested sample that prevented detectable metabolic reduction in resazurin compared to the growth control. For quantitative analysis, bacterial viability was expressed as a percentage relative to the untreated growth control after appropriate blank correction. IC_50_ values were calculated by nonlinear regression. All experiments were performed in triplicate.

### 3.7. Statistical Analysis

All the data are presented as mean ± standard deviation (SD) based on three independent experiments. Statistical analysis was performed using one-way analysis of variance (ANOVA), followed by Dunnett’s and Tukey’s post hoc tests. Statistical significance was defined as **** *p* < 0.0001, *** *p* < 0.001, ** *p* < 0.01, and * *p* < 0.05 relative to the control group. All analyses were conducted using GraphPad Prism software (version 8.4.3; GraphPad Software, Inc., San Diego, CA, USA).

## 4. Conclusions

This study examined the biological activity of unfermented and kombucha-fermented extracts obtained from the cores and leaves of white and red cabbage. The obtained results show that the analyzed ferments are characterized by increased levels of biologically active compounds compared to the unfermented extracts. Furthermore, it is worth emphasizing that better antioxidant, antibacterial, and anti-inflammatory properties were observed in the extracts and ferments from white and red cabbage leaves compared to the core. Analyses of the antioxidant properties showed that the ferments have a better ability to scavenge free radicals and reduce reactive oxygen species (ROS). This may be due to the higher phytochemical content in the ferments, which was confirmed by chromatographic analyses. Cytotoxicity studies show that low concentrations have a positive effect on cell viability, while higher concentrations (500 µg/mL) result in decreased viability. Moreover, the ferments were shown to have a greater ability to reduce the levels of the tested cytokines (IL-1B and IL-6) compared to the unfermented extracts. The obtained results clearly indicate that antibacterial efficacy was strongly dependent on fermentation time, plant part, cabbage variety, and bacterial species. The ferments demonstrated stronger antibacterial activity than the corresponding aqueous extracts, and these properties increased with time (F10 and F20). These findings confirm that fermentation effectively increases the antibacterial potential of raw materials derived from cabbage and improves their functional properties. The obtained results clearly confirm that fermentation using SCOBYs is a highly justified process, as it increases the bioactivity of waste materials compared to their starting aqueous extracts. The observed increase in antioxidant, anti-inflammatory, and antibacterial properties, reaching a maximum after 20 days of the process, demonstrates that biotransformation effectively unlocks the hidden potential of cabbage cores and leaves. Improving biological parameters makes fermentation a key tool for high-value waste management, enabling the transformation of low-value by-products into valuable, safe and ecological ingredients with a broad spectrum of action.

## Figures and Tables

**Table 1:** Quantitative phytochemical analysis of white cabbage extract (E) and its fermented samples after 10 days (F10) and 20 days (F20) of fermentation (µg/mL).

Compound	Kombucha Solution	White Cabbage Core			White Cabbage Leaf		
		E	F10	F20	E	F10	F20
Gallic acid	0.95 ± 0.06 ^c^	nd	1.96 ± 0.13 ^a^	1.98 ± 0.14 ^a^	nd	1.81 ± 0.12 ^b^	1.89 ± 0.13 ^ab^
Galloylquinic acids	0.62 ± 0.04 ^c^	nd	1.28 ± 0.09 ^b^	1.29 ± 0.09 ^b^	nd	1.27 ± 0.08 ^b^	1.38 ± 0.10 ^a^
Chlorogenic acids	0.17 ± 0.01 ^c^	nd	0.26 ± 0.02 ^b^	0.28 ± 0.02 ^b^	nd	0.36 ± 0.03 ^a^	0.37 ± 0.03 ^a^
Catechin/epicatechin	0.22 ± 0.02 ^c^	nd	0.95 ± 0.07 ^a^	1.01 ± 0.08 ^a^	nd	0.57 ± 0.04 ^b^	0.62 ± 0.05 ^b^
p-Coumaroylquinic acid isomers	0.46 ± 0.03 ^c^	nd	0.86 ± 0.06 ^b^	0.95 ± 0.07 ^a^	nd	0.88 ± 0.06 ^ab^	0.90 ± 0.06 ^ab^
Quercetin 3-O-glucoside	0.09 ± 0.01 ^a^	nd	0.07 ± 0.00 ^b^	0.02 ± 0.00 ^c^	nd	0.01 ± 0.00 ^c^	0.01 ± 0.00 ^c^
Kaempferol derivative *m*/*z* 593	0.29 ± 0.02 ^c^	nd	0.54 ± 0.04 ^b^	0.56 ± 0.04 ^b^	nd	0.59 ± 0.04 ^ab^	0.62 ± 0.05 ^a^
Kaempferol hexoside	0.07 ± 0.01 ^b^	nd	0.10 ± 0.01 ^a^	0.03 ± 0.00 ^c^	nd	0.02 ± 0.00 ^c^	0.01 ± 0.00 ^c^
Kaempferol derivative *m*/*z* 755	0.14 ± 0.01 ^c^	nd	0.28 ± 0.02 ^b^	0.34 ± 0.03 ^a^	nd	0.29 ± 0.02 ^b^	0.31 ± 0.02 ^ab^
Apigenin derivative *m*/*z* 577	0.16 ± 0.01 ^c^	nd	0.28 ± 0.02 ^ab^	0.29 ± 0.02 ^a^	nd	0.26 ± 0.02 ^b^	0.27 ± 0.02 ^b^
Flavonoid *m*/*z* 563	0.21 ± 0.01 ^b^	nd	0.44 ± 0.03 ^a^	0.45 ± 0.03 ^a^	nd	0.42 ± 0.03 ^a^	0.46 ± 0.03 ^a^
Quercetin 3-O-rutinoside	0.13 ± 0.01 ^c^	nd	0.63 ± 0.04 ^a^	0.67 ± 0.05 ^a^	nd	0.40 ± 0.03 ^b^	0.46 ± 0.03 ^b^
Kaempferol derivative *m*/*z* 609	nd	0.01 ± 0.00 ^d^	0.06 ± 0.00 ^c^	0.10 ± 0.01 ^b^	0.12 ± 0.01 ^b^	0.23 ± 0.02 ^a^	0.25 ± 0.02 ^a^
Quercetin derivative *m*/*z* 771	0.06 ± 0.00 ^c^	nd	0.12 ± 0.01 ^a^	0.13 ± 0.01 ^a^	nd	0.09 ± 0.01 ^b^	0.11 ± 0.01 ^ab^

Values are expressed as mean ± SD (*n* = 3). Different superscript letters within a row indicate statistically significant differences at *p* < 0.05 according to one-way ANOVA followed by Tukey’s post hoc test. “nd”—not detected.

**Table 2:** Quantitative phytochemical analysis of red cabbage extract (E) and its fermented samples after 10 days (F10) and 20 days (F20) of fermentation (µg/mL).

Compound	Kombucha Solution	Red Cabbage Core			Red Cabbage Leaf		
		E	F10	F20	E	F10	F20
Gallic acid	0.95 ± 0.06 ^c^	nd	1.32 ± 0.09 ^b^	1.36 ± 0.10 ^b^	nd	1.68 ± 0.12 ^a^	1.85 ± 0.13 ^a^
Galloylquinic acids	0.62 ± 0.04 ^c^	nd	1.04 ± 0.07 ^b^	1.12 ± 0.08 ^b^	nd	1.28 ± 0.09 ^a^	1.31 ± 0.10 ^a^
Chlorogenic acids	0.17 ± 0.01 ^c^	nd	0.24 ± 0.02 ^b^	0.25 ± 0.02 ^b^	nd	0.31 ± 0.02 ^a^	0.34 ± 0.03 ^a^
Catechin/epicatechin	0.22 ± 0.02 ^c^	nd	0.23 ± 0.02 ^c^	0.25 ± 0.02 ^c^	nd	0.34 ± 0.03 ^b^	0.63 ± 0.05 ^a^
p-Coumaroylquinic acid isomers	0.46 ± 0.03 ^c^	0.02 ± 0.00 ^d^	1.10 ± 0.08 ^a^	1.12 ± 0.08 ^a^	0.06 ± 0.00 ^d^	0.85 ± 0.06 ^b^	0.96 ± 0.07 ^b^
Quercetin 3-O-glucoside	0.09 ± 0.01 ^a^	nd	nd	0.01 ± 0.00 ^b^	nd	nd	0.03 ± 0.00 ^b^
Kaempferol derivative *m*/*z* 593	0.29 ± 0.02 ^c^	nd	0.59 ± 0.04 ^a^	0.56 ± 0.04 ^ab^	nd	0.53 ± 0.04 ^b^	0.59 ± 0.04 ^a^
Kaempferol hexoside	0.07 ± 0.01 ^a^	nd	nd	0.02 ± 0.00 ^c^	nd	0.01 ± 0.00 ^c^	0.05 ± 0.00 ^b^
Kaempferol derivative *m*/*z* 755	0.14 ± 0.01 ^c^	nd	0.29 ± 0.02 ^b^	0.32 ± 0.02 ^a^	nd	0.26 ± 0.02 ^b^	0.30 ± 0.02 ^ab^
Apigenin derivative *m*/*z* 577	0.16 ± 0.01 ^c^	nd	0.32 ± 0.02 ^b^	0.36 ± 0.03 ^a^	nd	0.30 ± 0.02 ^b^	0.39 ± 0.03 ^a^
Flavonoid *m*/*z* 563	0.21 ± 0.02 ^c^	nd	0.47 ± 0.03 ^a^	0.51 ± 0.04 ^a^	nd	0.41 ± 0.03 ^b^	0.42 ± 0.03 ^b^
Quercetin 3-O-rutinoside	0.13 ± 0.01 ^c^	0.01 ± 0.00 ^d^	0.22 ± 0.02 ^b^	0.34 ± 0.03 ^b^	nd	0.32 ± 0.02 ^b^	0.56 ± 0.04 ^a^
Kaempferol derivative *m*/*z* 609	nd	nd	0.03 ± 0.00 ^c^	0.04 ± 0.00 ^c^	0.02 ± 0.00 ^c^	0.06 ± 0.00 ^b^	0.07 ± 0.01 ^a^
Quercetin derivative *m*/*z* 771	0.06 ± 0.00 ^c^	nd	0.08 ± 0.01 ^b^	0.09 ± 0.01 ^b^	nd	0.09 ± 0.01 ^b^	0.18 ± 0.01 ^a^

Values are expressed as mean ± SD (*n* = 3). Different superscript letters within a row indicate statistically significant differences at *p* < 0.05 according to one-way ANOVA followed by Tukey’s post hoc test. “nd”—not detected.

**Table 3:** Minimum inhibitory concentration (MIC, µg/mL) of cabbage extracts (E) and kombucha-fermented preparations (F10—10-day fermentation; F20—20-day fermentation) from core and outer leaves of white and red cabbage against selected bacterial strains.

Bacteria	Minimum Inhibitory Concentration MIC [μg/mL]											
	White Cabbage						Red Cabbage					
	Core			Leaf			Core			Leaf		
	E	F10	F20	E	F10	F20	E	F10	F20	E	F10	F20
*Staphylococcus aureus*	484	342	260	>500	433	360	420	384	302	395	210	154
*Staphylococcus capitis*	>500	428	340	451	387	325	460	415	385	380	264	215
*Micrococcus luteus*	475	402	305	>500	420	415	415	380	357	380	322	230
*Escherichia coli*	>500	422	364	>500	405	384	>500	422	355	445	388	305
*Pseudomonas aeruginosa*	>500	402	368	>500	>500	435	>500	>500	450	>500	452	383

## Data Availability

The data presented in this study are available on request from the corresponding author.

## Supplementary Materials

The following supporting information can be downloaded at https://www.mdpi.com/article/10.3390/molecules31111886/s1, Figure S1. Representative base peak chromatograms (BPCs) and DAD chromatograms recorded at λ = 320 nm for white cabbage core extract (green line) and fermented extracts after 10 days (red line) and 20 days (blue line) of fermentation; Figure S2. Representative extracted ion chromatograms (EICs) of amino acids showing changes in (a) aspartic acid and (b) glutamic acid in the extract (green line), after 10 days of fermentation (red line), and after 20 days of fermentation (blue line); Figure S3. Representative extracted ion chromatograms (EICs) of low-molecular-weight organic acids showing changes in (a) citric acid and (b) malic acid in the extract (green line), after 10 days of fermentation (red line), and after 20 days of fermentation (blue line); Figure S4. The ability of kombucha to scavenge ABTS free radicals (A), scavenge DPPH free radicals (B) and reduce ferric ions in FRAP assay (C) at concentrations of 50, 100, 250 and 500 µg/mL. Data are presented as mean ± SD from three independent experiments, with each sample tested in triplicate. **** *p* < 0.0001, ** *p* = 0.0066; Figure S5. The ability of kombucha on the intracellular level of reactive oxygen species in fibroblasts (HDFs), at concentrations of 50, 100, 250 and 500 µg/mL. Cells cultured in a medium without the tested extracts served as the negative control (NC), while cells stimulated with hydrogen peroxide (H_2_O_2_) were used as the positive control (PC). Data are presented as mean ± SD from three independent experiments, with each sample tested in triplicate **** *p* < 0.0001; Figure S6. Effect of the increase in the concentration of kombucha (50–500 μg/mL) on cell viability (Alamar Blue assay) by cultured fibroblasts (A) and keratinocytes (B) and on cell viability (Neutral Red assay) by cultured fibroblasts (C) and karatinocytes (D). Data are the mean ± SD of three independent experiments each consisting of three replicates per test group. ** *p* < 0.01; Figure S7. The impact of kombucha on IL-1β levels (A) and IL-6 levels (B) in THP-1 cells stimulated with bacterial LPS (10 μg/mL), expressed as fold compared to the negative control (NC). The positive control (PC) consisted of cells stimulated with LPS but without the addition of E, F10 and F20. Diclofenac (D; 10 µg/mL) was used as reference compound. Data represents the mean ± SD of three independent experiments, with each sample tested in duplicate. **** *p* < 0.0001; Table S1. Representative MS data for components identified in white cabbage extracts from different parts of plants including core (WCC) and leaves (WCL) and in the extracts obtained after 20 days of fermentation (F20); Table S2. Quantitative analysis of amino acids in white cabbage (µg/mL); Table S3. Quantitative analysis of amino acids in red cabbage (µg/mL); Table S4. Quantitative analysis of low molecular acids in white cabbage [µg/g]; Table S5. Quantitative analysis of low molecular acids in red cabbage [µg/g].

## Abbreviations

The following abbreviations are used in this manuscript:

AB	Alamar Blue Assay
ABTS	2,2′-azino-bis(3-ethylbenzothiazoline-6-sulfonic acid)
AHA	alpha hydroxy acids
CFU	colony forming units
CO_2_	carbon dioxide
COX-2	cyclooxygenase 2
DAD	diode array detector
DCF	2′,7′-dichlorofluorescein
DMEM	dulbecco’s modified eagle medium
DPPH	1,1-diphenyl-2-picrylhydrazyl
E	extract
ELISA	enzyme-linked immunosorbent assay
ESI/TOF	electrospray ionization/time-of-flight
F10	10-day ferment
F20	20-day ferment
FBS	fetal bovine serum
FRAP	ferric reducing antioxidant power
H_2_DCFDA	2′,7′-dichlorodihydrofluorescein diacetate
H_2_O_2_	hydrogen peroxide
HaCaT	human keratinocyte cell line
HDF	human dermal fibroblasts
IL-10	interleukin 10
IL-1β	interleukin-1 beta
IL-6	interleukin 6
iNOS	inducible nitric oxide synthase
LPS	lipopolysaccharide
MIC	minimum inhibitory concentration
NF-κB	nuclear factor kappa B
NO	nitric oxide
NR	Neutral Red Assay
PBS	phosphate-buffered saline
RAW264.7	murine macrophage cell line
ROS	reactive oxygen species
SCOBY	symbiotic culture of bacteria and yeast
TEWL	transepidermal water loss
THP-1	human acute monocytic leukemia cell line
TNF-α	tumor necrosis factor alpha
TPTZ	2,4,6-Tripyridyl-s-triazine
UHPLC	ultra-high-performance liquid chromatography
UV-VIS	ultraviolet–visible spectroscopy
