# Supplementary material for: Kombucha Ferments from White and Red Cabbage By-Products as a Sustainable Source of Metabolites with Antioxidant and Anti-Inflammatory Activity
Source: Molecules. 2026 Jun 1;31(11):1886. doi: 10.3390/molecules31111886 (PMC13257639; doi:10.3390/molecules31111886)
Supplement: Supplementary file 1 [file molecules-31-01886-s001.zip › molecules-4311312-supplementary.pdf]

*molecules*

*Article*

# Kombucha fermentation as a sustainable approach for valorization of cabbage by-products into value-added bioactive ingredients

**Zofia Nizioł-Łukaszewska[1], Aleksandra Ziemlewska[1], Agnieszka Mokrzyńska[1], Magdalena Wójciak[2], Ireneusz Sowa[2], Martyna Zagórska-Dziok[1,*]**

1 Department of Technology of Cosmetic and Pharmaceutical Products, Medical College, University of Information Technology and Management in Rzeszow, Sucharskiego 2, 35-225 Rzeszow, Poland
2 Department of Analytical Chemistry, Medical University of Lublin, Aleje Raclawickie 1, 20-059 Lublin, Poland
* Correspondence: mzagorska@wsiz.edu.pl

Academic Editor: Arjun H. Banskota

Received: 25 April 2026
Revised: 20 May 2026
Accepted: 27 May 2026
Published: 1 June 2026

**Copyright:** © 2026 by the author. Licensee MDPI, Basel, Switzerland. This article is an open access article distributed under the terms and conditions of the Creative Commons Attribution (CC BY) license.

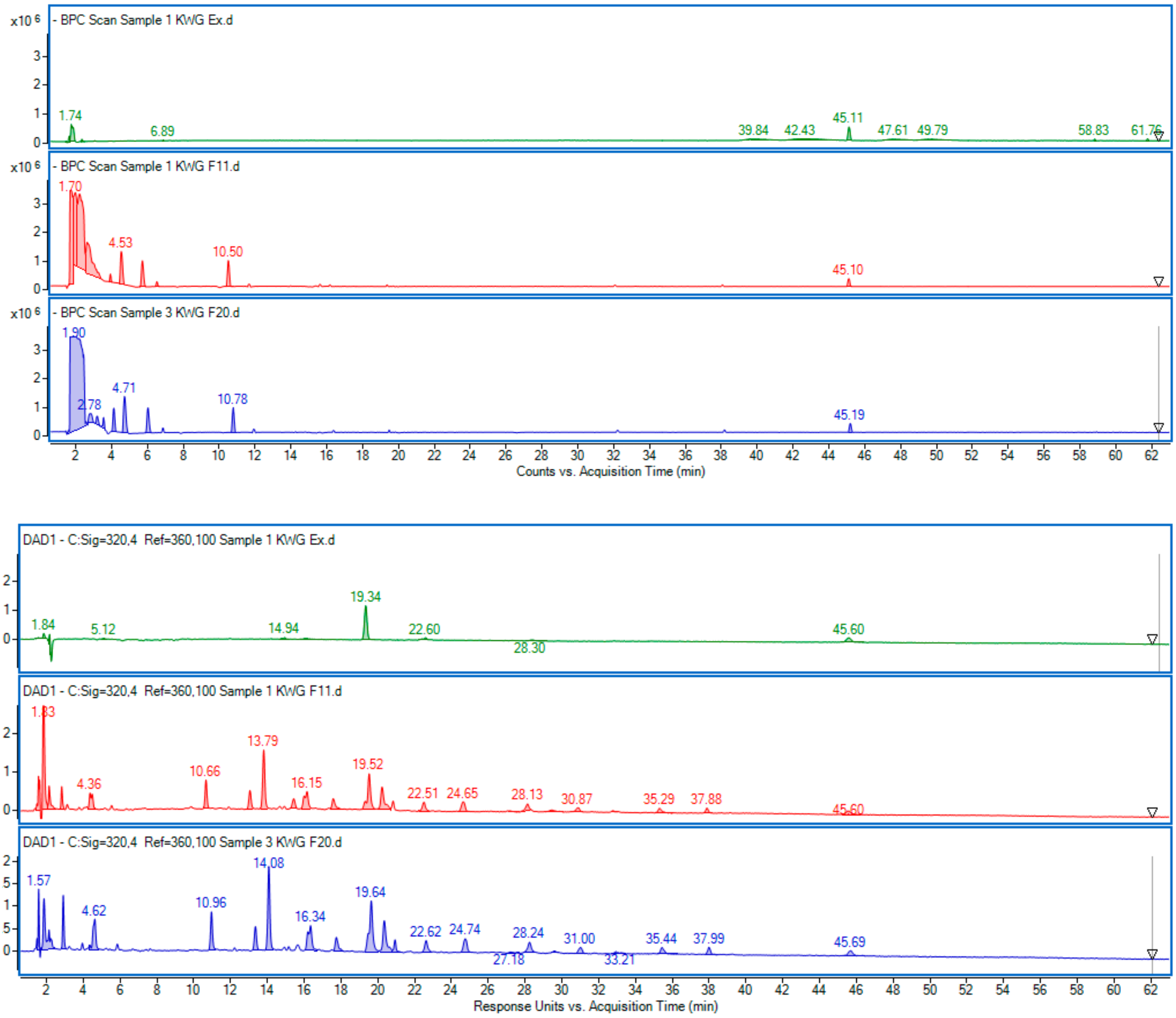


**Figure S1**. Representative base peak chromatograms (BPCs) and DAD chromatograms recorded at $\lambda$ = 320 nm for white cabbage core extract (green line) and fermented extracts after 10 days (red line) and 20 days (blue line) of fermentation.

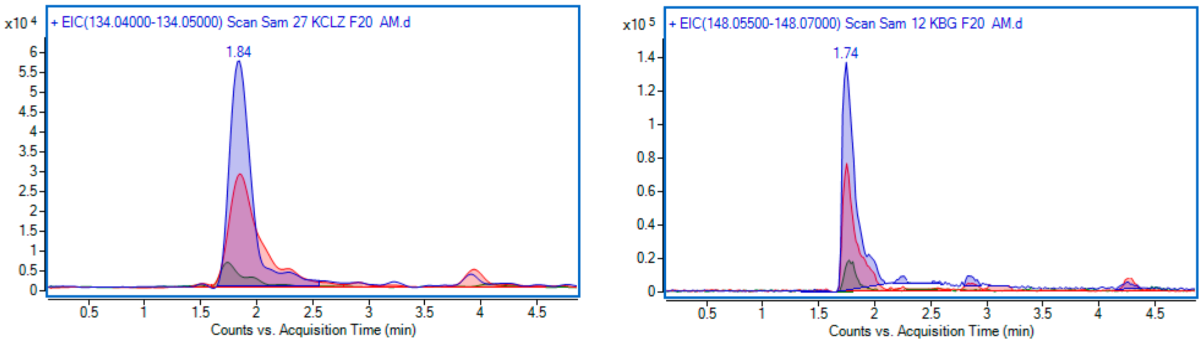


**Figure S2.** Representative extracted ion chromatograms (EICs) of amino acids showing changes in (a) aspartic acid and (b) glutamic acid in the extract (green line), after 10 days of fermentation (red line), and after 20 days of fermentation (blue line).

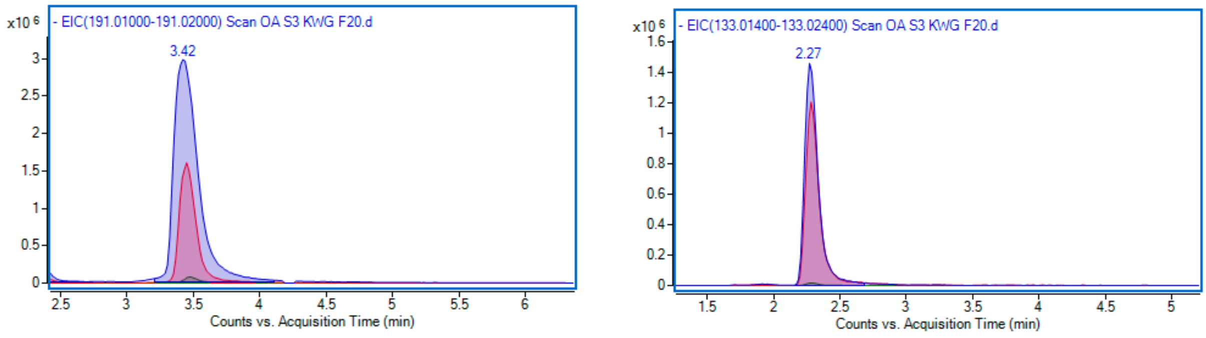


**Figure S3.** Representative extracted ion chromatograms (EICs) of low-molecular-weight organic acids showing changes in (a) citric acid and (b) malic acid in the extract (green line), after 10 days of fermentation (red line), and after 20 days of fermentation (blue line).

**Table S1**. Representative MS data for components identified in white cabbage extracts from different parts of plants including core (WCC) and leaves (WCL) and in the extracts obtained after 20 days of fermentation (F20).

| Rt (min) | [m/z-H]- | Error (ppm) | Compound | WCC | WCCF20 | WCL | WCL F20 |
|---|---|---|---|---|---|---|---|
| 4.53 | 169.01499 | 4.37 | Galic Acid* | - | + | - | + |
| 5.72 | 343.06602 (169,191) | -3.05 | Galloylquinic acid | - | + | - | + |
| 6.51 | 343.06559 (169,191) | -4.30 | Galloylquinic acid | - | + | - | + |
| 10.98 | 353.08823 (191, 179) | 1.20 | Neochlorogenic acid* | - | + | - | + |
| 13.23 | 337.09156(163, 191,173) | -3.94 | 3-p-Coumaroylquinic acid (I) | - | + | - | + |
| 13.98 | 337.09187 (163, 191,173) | -3.02 | 3-p-Coumaroylquinic acid (II)* | - | + | - | + |
| 15.84 | 289.07128 (245,221) | -1.66 | Catechin | - | + | - | + |
| 16.13 | 353.08831 (191, 179) | 1.42 | Chlorogenic acid* | + | + | + | + |
| 17.77 | 337.09201 (191,173) | -2.61 | 4-p-Coumaroylquinic acid (I) | - | + | - | + |
| 19.70 | 337.09227 (191,173) | -1.84 | 4-p-Coumaroylquinic acid (II)* | - | + | - | + |
| 19.72 | 289.07109 (245,221) | -2.32 | Epicatechin* | - | + | - | + |
| 20.40 | 337.09199 (191,173) | -2.67 | 5-p-Coumaroylquinic acid (I) | - | + | - | + |
| 22.67 | 337.09285 (191,173) | -0.12 | 5-p-Coumaroylquinic acid (II)* | - | + | - | + |
| 24.82 | 563.14065 | 0.04 | Unknown flavonoid | - | + | - | + |
| 28.32 | 593.15344 | 3.78 | Kaempferol derivative | - | + | - | + |
| 29.64 | 577.15631 | 0.05 | Apigenin derivative | - | + | - | + |
| 30.04 | 771.19826 | -0.87 | Quercetin derivative | - | + | - | + |
| 30.65 | 609.14781 | 2.79 | Kaempferol derivative | + | + | + | + |
| 31.11 | 577.15694 | 1.14 | Apigenin derivative | - | + | - | + |
| 32.07 | 609.14711 (463,300) | 1.64 | Quercetin 3-O-rutinoside* | - | + | - | + |
| 33.06 | 755.20304 | -1.29 | Kaempferol derivative | - | + | - | + |
| 33.5 | 463.09003 (300) | 3.94 | Quercetin 3-O-glucoside* | - | + | - | + |
| 35.60 | 755.20395 | -0.09 | Kaempferol derivative | - | + | - | + |

| | | | | | | | |
|---|---|---|---|---|---|---|---|
| 37.22 | 447.09399 | 1.57 | Kaempferol hexoside | - | + | - | + |
| 38.05 | 593.15047 | -1.22 | Kaempferol 3-O-rutinoside* | - | + | - | + |
| 39.32 | 447.09356 | 0.61 | Kaempferol 3-O-glucoside * | - | + | - | + |

* confirmed by comparison with standard

**Table S2.** Quantitative analysis of amino acids in white cabbage (μg/mL).

| Compound | Kombucha solution | White Cabbage core | | | White Cabbage leaf | | |
|---|---|---|---|---|---|---|---|
| | | E | F10 | F20 | E | F10 | F20 |
| Aspartic acid | 0.11 ± 0.01$^{c}$ | 0.04 ± 0.00$^{d}$ | 0.21 ± 0.02$^{b}$ | 0.36 ± 0.03$^{a}$ | 0.01 ± 0.00$^{d}$ | 0.24 ± 0.02$^{b}$ | 0.40 ± 0.03$^{a}$ |
| Glutamine | 0.08 ± 0.01$^{c}$ | 0.11 ± 0.01$^{c}$ | 0.41 ± 0.03$^{b}$ | 0.59 ± 0.04$^{a}$ | 0.02 ± 0.00$^{d}$ | 0.07 ± 0.01$^{c}$ | 0.16 ± 0.01$^{b}$ |
| Glutamic acid | 0.01 ± 0.00$^{d}$ | 0.09 ± 0.01$^{c}$ | 0.47 ± 0.03$^{b}$ | 0.67 ± 0.05$^{a}$ | 0.01 ± 0.00$^{d}$ | 0.02 ± 0.00$^{d}$ | 0.05 ± 0.00$^{c}$ |
| Proline | 0.02 ± 0.00$^{c}$ | 0.06 ± 0.00$^{c}$ | 1.01 ± 0.07$^{b}$ | 1.51 ± 0.11$^{a}$ | 0.02 ± 0.00$^{c}$ | 0.03 ± 0.00$^{c}$ | 0.04 ± 0.00$^{c}$ |
| Valine/norvaline | 0.35 ± 0.03$^{c}$ | 0.32 ± 0.02$^{c}$ | 0.56 ± 0.04$^{b}$ | 0.75 ± 0.05$^{a}$ | 0.04 ± 0.00$^{d}$ | 0.64 ± 0.05$^{b}$ | 0.92 ± 0.07$^{a}$ |
| Isoleucine/leucine | nd | 0.11 ± 0.01$^{c}$ | 0.25 ± 0.02$^{b}$ | 0.39 ± 0.03$^{a}$ | nd | 0.01 ± 0.00$^{d}$ | 0.01 ± 0.00$^{d}$ |

Values are expressed as mean ± SD (n = 3). Different superscript letters within a row indicate statistically significant differences at $p < 0.05$ according to one-way ANOVA followed by Tukey's post hoc test. "nd"—not detected.

**Table S3.** Quantitative analysis of amino acids in red cabbage (μg/mL).

| Compound | Kombucha solution | Red Cabbage core | | | Red Cabbage leaf | | |
|---|---|---|---|---|---|---|---|
| | | E | F10 | F20 | E | F10 | F20 |
| Aspartic acid | 0.11 ± 0.01$^{c}$ | 0.04 ± 0.00$^{d}$ | 0.29 ± 0.02$^{b}$ | 0.91 ± 0.07$^{a}$ | 0.01 ± 0.00$^{d}$ | 0.17 ± 0.01$^{c}$ | 0.44 ± 0.03$^{b}$ |
| Glutamine | 0.08 ± 0.01$^{c}$ | 0.01 ± 0.00$^{d}$ | 0.21 ± 0.02$^{b}$ | 0.23 ± 0.02$^{b}$ | 0.01 ± 0.00$^{d}$ | 0.17 ± 0.01$^{b}$ | 0.18 ± 0.01$^{b}$ |
| Glutamic acid | 0.01 ± 0.00$^{c}$ | 0.12 ± 0.01$^{a}$ | 0.03 ± 0.00$^{b}$ | 0.02 ± 0.00$^{bc}$ | 0.04 ± 0.00$^{b}$ | 0.03 ± 0.00$^{b}$ | 0.02 ± 0.00$^{bc}$ |
| Proline | 0.02 ± 0.00$^{c}$ | 0.10 ± 0.01$^{a}$ | 0.08 ± 0.01$^{ab}$ | 0.05 ± 0.00$^{b}$ | 0.02 ± 0.00$^{c}$ | 0.01 ± 0.00$^{c}$ | 0.03 ± 0.00$^{c}$ |
| Valine/norvaline | 0.35 ± 0.03$^{c}$ | 3.39 ± 0.24$^{a}$ | 1.38 ± 0.10$^{b}$ | 1.11 ± 0.08$^{b}$ | 0.15 ± 0.01$^{c}$ | 0.87 ± 0.06$^{b}$ | 1.15 ± 0.08$^{b}$ |
| Isoleucine/leucine | nd | 0.20 ± 0.02$^{a}$ | 0.01 ± 0.00$^{c}$ | 0.04 ± 0.00$^{b}$ | 0.01 ± 0.00$^{c}$ | 0.01 ± 0.00$^{c}$ | 0.01 ± 0.00$^{c}$ |

Values are expressed as mean ± SD (n = 3). Different superscript letters within a row indicate statistically significant differences at $p < 0.05$ according to one-way ANOVA followed by Tukey's post hoc test. "nd"—not detected.

**Table S4**. Quantitative analysis of low molecular acids in white cabbage [μg/g].

| Compound | Kombucha solution | White Cabbage core | | | White Cabbage leaf | | |
|---|---|---|---|---|---|---|---|
| | | E | F10 | F20 | E | F10 | F20 |
| Lactic acid | 1.4 ± 0.10[d] | 2.0 ± 0.14[c] | 6.9 ± 0.48[b] | 11.5 ± 0.81[a] | 2.5 ± 0.18[c] | 6.3 ± 0.44[b] | 14.8 ± 1.04[a] |
| Malic acid | 7.2 ± 0.50[c] | 1.0 ± 0.07[d] | 20.6 ± 1.44[b] | 51.5 ± 3.61[a] | 0.1 ± 0.01[e] | 9.5 ± 0.67[c] | 18.1 ± 1.27[b] |
| Citric acid | 5.2 ± 0.36[d] | 5.7 ± 0.40[d] | 45.2 ± 3.16[b] | 107 ± 7.49[a] | 1.2 ± 0.08[e] | 25.2 ± 1.76[c] | 51.6 ± 3.61[b] |

Values are expressed as mean ± SD (n = 3). Different superscript letters within a row indicate statistically significant differences at $p < 0.05$ according to one-way ANOVA followed by Tukey's post hoc test. "nd"—not detected.

**Table S5**. Quantitative analysis of low molecular acids in red cabbage [μg/g].

| Compound | Kombucha solution | Red Cabbage core | | | Red Cabbage leaf | | |
|---|---|---|---|---|---|---|---|
| | | E | F10 | F20 | E | F10 | F20 |
| Lactic acid | 1.4 ± 0.10[d] | 1.5 ± 0.11[d] | 6.8 ± 0.48[b] | 12.6 ± 0.88[a] | 1.9 ± 0.13[c] | 7.4 ± 0.52[b] | 11.3 ± 0.79[a] |
| Malic acid | 7.2 ± 0.50[c] | 0.3 ± 0.02[e] | 18.2 ± 1.27[b] | 33.9 ± 2.37[a] | 0.8 ± 0.06[d] | 10.2 ± 0.71[c] | 14.4 ± 1.01[b] |
| Citric acid | 5.2 ± 0.36[e] | 1.8 ± 0.13[f] | 22.5 ± 1.58[d] | 41.7 ± 2.92[c] | 2.3 ± 0.16[f] | 60.1 ± 4.21[b] | 107 ± 7.49[a] |

Values are expressed as mean ± SD (n = 3). Different superscript letters within a row indicate statistically significant differences at $p < 0.05$ according to one-way ANOVA followed by Tukey's post hoc test. "nd"—not detected.

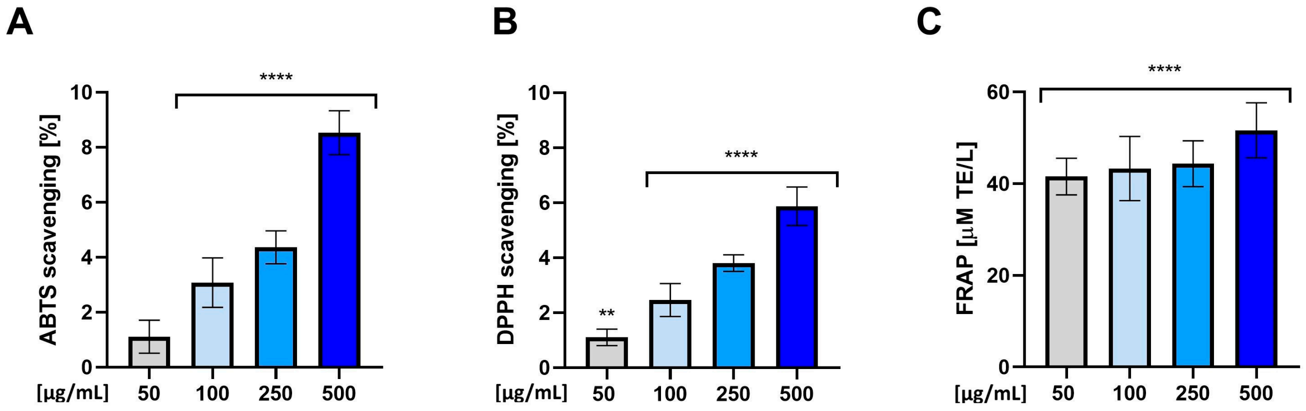


**Figure S4**. The ability of kombucha to scavenge ABTS free radicals (A), scavenge DPPH free radicals (B) and reduce ferric ions in FRAP assay (C) at concentrations of 50, 100, 250 and 500 μg/mL. Data are presented as mean ± SD from three independent experiments, with each sample tested in triplicate. **** $p < 0.0001$, ** $p = 0.0066$.

https://doi.org/10.3390/molecules31111886

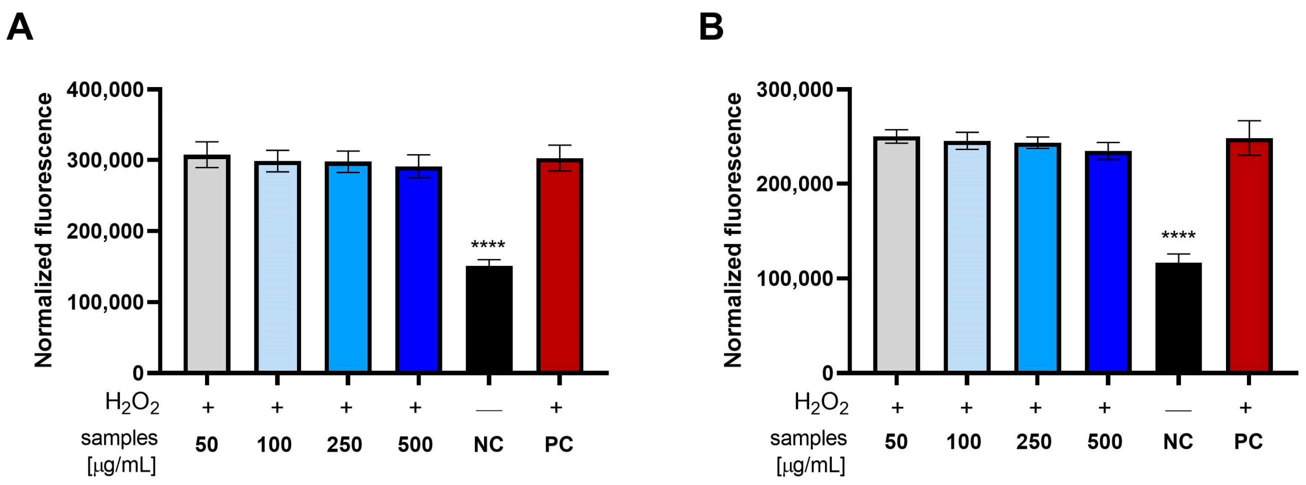


Figure S5. The ability of kombucha on the intracellular level of reactive oxygen species in fibroblasts (HDFs), at concentrations of 50, 100, 250 and 500 µg/mL. Cells cultured in a medium without the tested extracts served as the negative control (NC), while cells stimulated with hydrogen peroxide ($H_2O_2$) were used as the positive control (PC). Data are presented as mean ± SD from three independent experiments, with each sample tested in triplicate **** $p < 0.0001$.

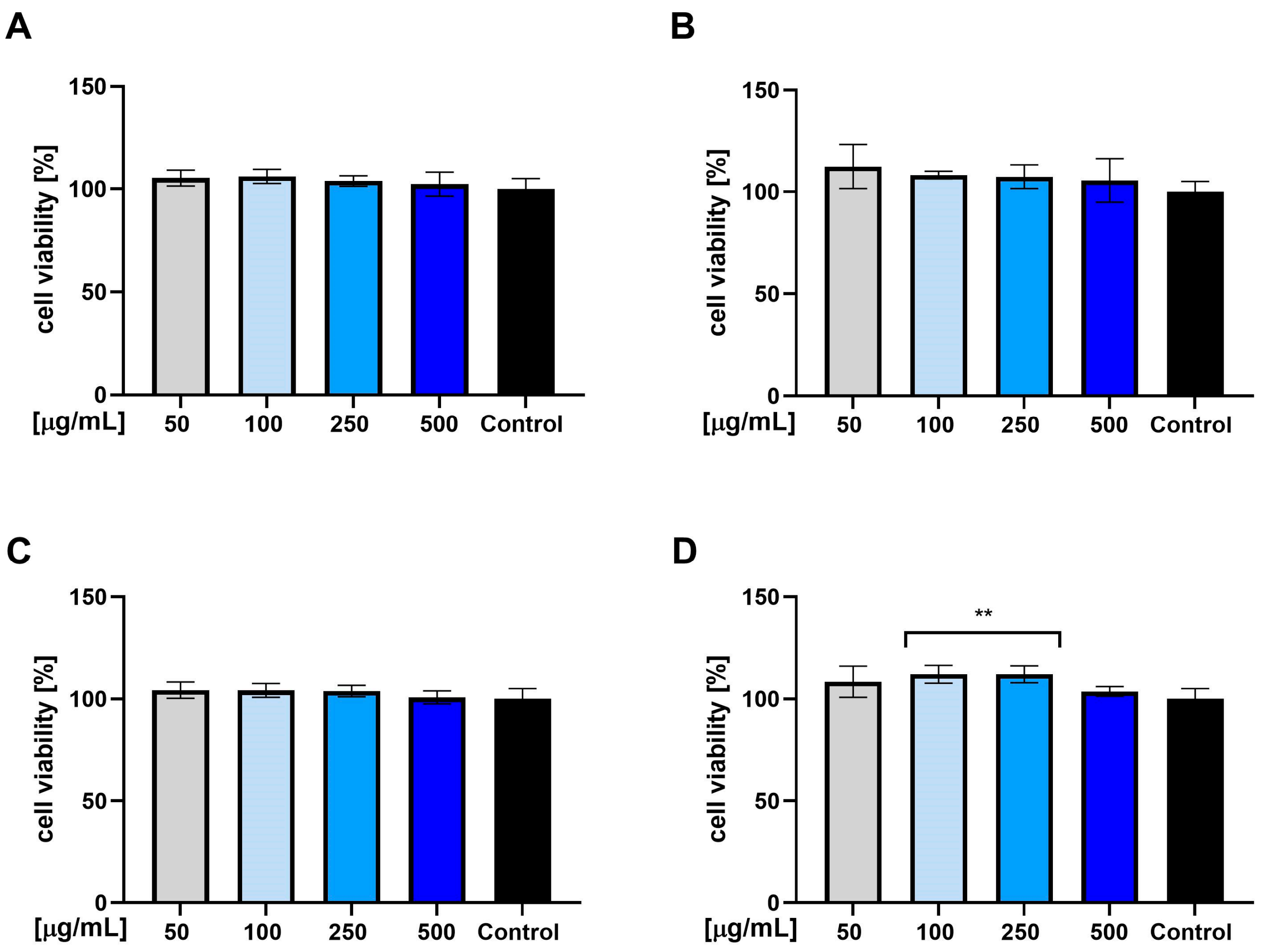


**Figure S6.** Effect of the increase in the concentration of kombucha (50 - 500 µg/mL) on cell viability (Alamar Blue assay) by cultured fibroblasts (A) and keratinocytes (B) and on cell viability (Neutral Red assay) by cultured fibroblasts (C) and karatinocytes (D). Data are the mean ± SD of three independent experiments each consisting of three replicates per test group. ** $p < 0.01$.

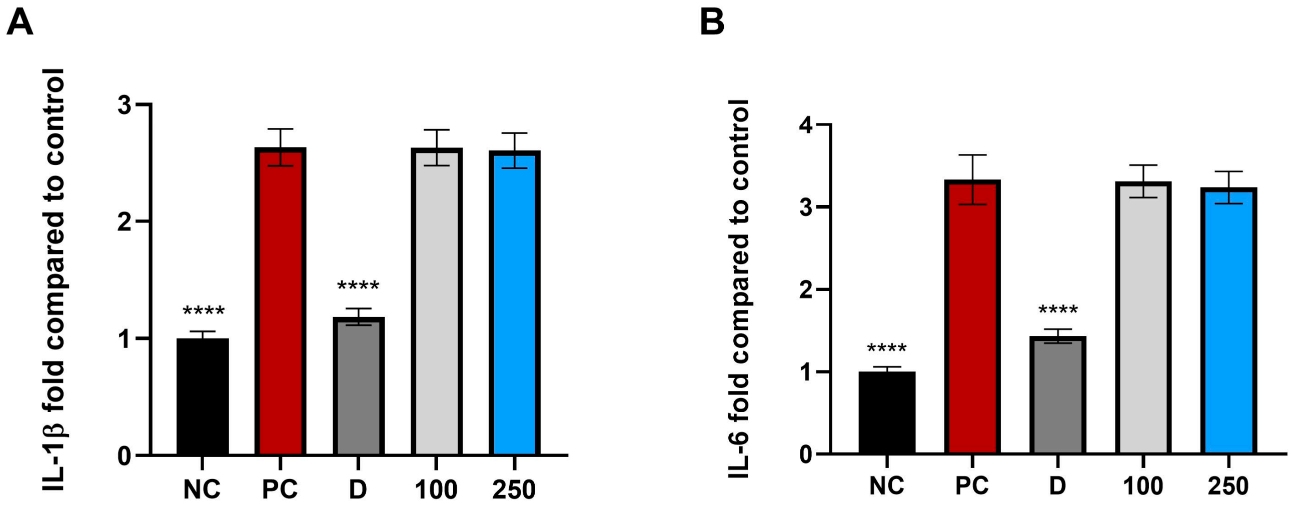


**Figure S7**. The impact of kombucha on IL-1β levels (A) and IL-6 levels (B) in THP-1 cells stimulated with bacterial LPS (10 μg/mL), expressed as fold compared to the negative control (NC). The positive control (PC) consisted of cells stimulated with LPS but without the addition of E, F10 and F20. Diclofenac (D; 10 μg/mL) was used as reference compound. Data represents the mean ± SD of three independent experiments, with each sample tested in duplicate. **** $p < 0.0001$.

https://doi.org/10.3390/molecules31111886
